# MicroRNA-mediated target mRNA cleavage and 3′-uridylation in human cells

**DOI:** 10.1038/srep30242

**Published:** 2016-07-21

**Authors:** Kai Xu, Jing Lin, Roza Zandi, Jack A. Roth, Lin Ji

**Affiliations:** 1 Section of Thoracic Molecular Oncology, Department of Thoracic and Cardiovascular Surgery, The University of Texas MD Anderson Cancer Center, 1515 Holcombe Boulevard, Houston, Texas 77030, USA

## Abstract

MicroRNAs (miRNAs) play an important role in targeted gene silencing by facilitating posttranscriptional and translational repression. However, the precise mechanism of mammalian miRNA-mediated gene silencing remains to be elucidated. Here, we used a stem-loop array reverse-transcription polymerase chain reaction assay to analyse miRNA-induced mRNA recognition, cleavage, posttranscriptional modification, and degradation. We detected endogenous let-7 miRNA-induced and Argonaute-catalysed endonucleolytic cleavage on target mRNAs at various sites within partially paired miRNA:mRNA sequences. Most of the cleaved mRNA 5′-fragments were 3′-oligouridylated by activities of terminal uridylyl transferases (TUTases) in miRNA-induced silencing complexes and temporarily accumulated in the cytosol for 5′-3′ degradation or other molecular fates. Some 3′-5′ decayed mRNA fragments could also be captured by the miRNA-induced silencing complex stationed at the specific miRNA:mRNA target site and oligouridylated by other TUTases at its proximity without involving Argonaute-mediated RNA cleavage. Our findings provide new insights into the molecular mechanics of mammalian miRNA-mediated gene silencing by coordinated target mRNA recognition, cleavage, uridylation and degradation.

MicroRNAs (miRNAs) are noncoding RNAs that have been shown to posttranscriptionally regulate gene expression and protein synthesis, particularly in mammalian cells, by partially base-pairing to complementary sequences in the 3′-untranslated regions (3′UTRs) of their target mRNAs^1^,^2^,^3^. Mature miRNAs are incorporated into Argonaute (Ago) proteins and serve as guide molecules in miRNA-induced silencing complexes (miRISCs) for target-specific gene silencing^4^,^5^. The miRNA-directed destruction of target mRNAs through Ago-catalysed mRNA cleavage has been shown to be a dominant model of repression of gene expression in plants and of short-interfering RNA (siRNA) action in eukaryotes, in which miRNAs or siRNAs pair to their mRNA targets extensively to ensure irreversible cleavage of the mRNAs^6^. Although mammalian miRNA has recently been shown to predominantly decrease target mRNA levels, leading to reduced protein output^7^, direct evidence for miRNA-mediated target mRNA cleavage and degradation has not been fully demonstrated in mammalian cells because extensive miRNA:target base-pairing is rare there^8^. Mammalian miRNA recognizes its mRNA targets with much shorter base pairs, usually located at the evolutionarily conserved nucleotide (nt) position 2–7 at the 5′ termini of miRNAs with compensatory support from the 3′ supplementary base-pairing^1^. The cleavage on mouse endogenous *HOX B8* mRNA, directed by the perfectly base-matched miR-196, is one of only a few cases of miRNA-directed mRNA decay reported in mammalian cells^9^. Moreover, a few mRNA cleavages were also mapped to miRNA target sites with near-perfect miRNA:target pairings by RNA-deep sequencing^10^,^11^,^12^. However, owing to the limited cases of detectable mRNA cleavage and the prerequisite of extensive base-pairing between miRNAs and their mRNA targets, mammalian miRNA-mediated mRNA cleavage has been regarded as exceptional rather than a general rule in the mechanism of gene silencing.

It is very important to provide direct evidence for miRNA-mediated target mRNA cleavage and degradation to access the precise mechanism of action in the miRNA-mediated suppression of target gene expression in mammalian cells. The techniques currently used to study the individual or global effects of miRNA action on its target mRNA, such as 5′RACE, ribosome profiling, and RNA-deep sequencing, are powerful and informative^2^,^8^,^10^,^13^, but these techniques are limited to investigation of the steady-state effects of miRNA activities and lack the sensitivity and specificity to capture dynamic changes of the short-lived mRNA intermediates resulting from miRNA activities under physiological conditions^14^. Here, we used a stem-loop array reverse-transcription polymerase chain reaction (SLA–RT-PCR) assay^14^ derived from a method developed by Chen *et al*.^15^ to detect the specific 5′-RNA fragments cleaved by mammalian miRNA activities on the basis of their 3′ terminal sequences to identify accurate cleavage sites on the target mRNAs. Using this approach, we detected miRNA-mediated mRNA cleavage activities at various sites within the predicted miRNA:mRNA partial base-pairing sequences and in their 5′- and 3′-adjacent regions, as well as posttranscriptional oligouridyl modifications at the 3′-termini of cleaved mRNA 5′-fragments at different time points of miRNA action under physiological conditions.

## Results

### miRNA Let-7–targeted *TUSC2* mRNA Cleavage

**Figure 1:**
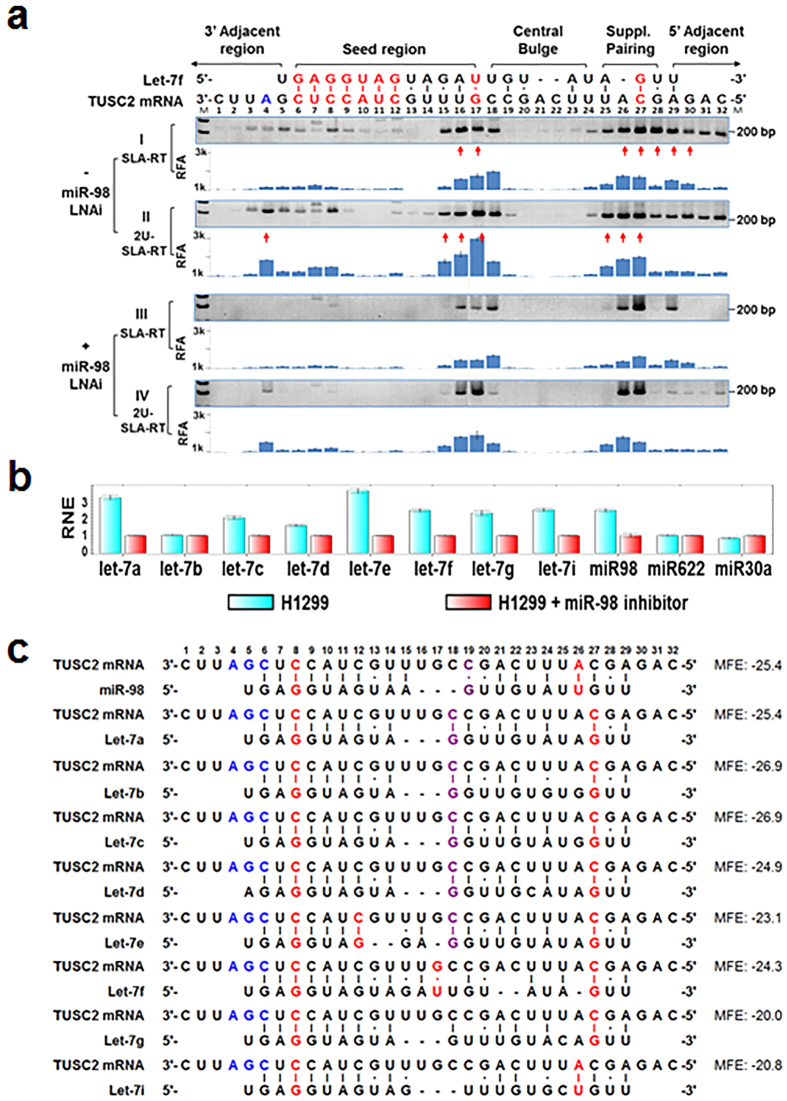
Prediction and detection of the let-7 miRNA–mediated target cleavage sites in the 3′UTR of *TUSC2* mRNA. (**a**) Cleaved mRNA 5′-fragments were detected by SLA–RT-PCR assay. Total RNAs were prepared from H1299 human lung cancer cells 48 h after treatment with or without the miR-98-LNA inhibitor **(+/−miR-98-LNAi**). RT was performed using SLA-RT primers specific to the 3′-end sequences of 5′-fragments of all the possible mRNA cleavage intermediates across the predicted let-7 miRNA:*TUSC2* mRNA binding site (including seed, central bulge, and supplementary pairing regions) and 3′- and 5′-adjacent regions, as depicted. The SLA-RT products were converted into cDNA and detected by subsequent PCR amplification. The specific SLA–RT-PCR products with predicted sizes ranging from 232 bp to 200 bp in the direction from 3′ to 5′ on target *TUSC2* mRNA sequences were separated on 2% agarose gel (**M**: 0.03 μg of 1 kb DNA ladder) or quantified by qRT-PCR as demonstrated by the relative fragment abundance (RFA) on qRT-PCR histograms. The sequence-verified SLA–RT-PCR fragments are indicated by red arrows. The SLA–RT-PCR amplicon intensity with the predicted size in each lane represents the abundance of the potentially cleaved *TUSC2* mRNA 5′-fragments with and without 3′-uridylation and was detected by the designated SLA-RT and 2U-SLA-RT primers, respectively (primer details in Supplementary Table 1). (**b**) Relative normalised expression (RNE) level of *let-7* miRNA family members in H1299 cells treated or not treated with miR-98-LNAi was detected by real-time SLA–qRT-PCR. *miR-622* and *miR-30a* were used as nonspecific controls and their expression levels were normalised to those of RNU44. SLA-RT primers and PCR primers are listed in Supplementary Table 3. (**c**) *let-7* miRNA:*TUSC2* target mRNA sequence pairing and potential cleavage sites were detected by a minimal free energy (MFE)–based miRmate algorithm^17^. The nt at the predicted and detected possible cleavage sites with the lowest MFE, cleavage blockage sites and mix of both are marked as red, blue, and purple, respectively.

We used the most extensively characterised human miRNAs, the let-7 family, and their natural mRNA target, the tumour suppressor candidate 2 gene (*TUSC2*, also known as *FUS1*), to demonstrate the mammalian miRNA-mediated target mRNA cleavage and regulatory activities in human cells using a novel SLA–RT-PCR assay (Fig. 1a). *TUSC2* mRNA is constitutively expressed in human non-small cell lung cancer H1299 cells^16^. Varied expression levels of the let-7 family miRNAs were detected in H1299 cells by real-time quantitative RT-PCR (qRT-PCR) (Fig. 1b). We previously identified miR-98, a member of the let-7 miRNA family, as targeting the 3′UTR of *TUSC2* mRNA and showed that overexpression of miR-98 decreased the *TUSC2* mRNA level in various NSCLC cells^17^.

To further understand the molecular mechanism of miR-98–induced downregulation of *TUSC2* expression, we used the SLA–RT-PCR assay to detect the possible accumulation of cleaved 5′-fragments of *TUSC2* mRNA (Fig. 1a). A series of SLA-RT primers with a 6-nt probe at their 3′ termini was designed to match along the entire let-7:*TUSC2* mRNA target sequence as well as their 5′- and 3′-adjacent regions for the initial RT reaction (Supplementary Fig. 1a and Supplementary Table 1a), using total RNAs prepared from H1299 cells (Fig. 1a-I,II) as RT templates. A cleaved 5′-mRNA fragment with its 3′ terminal sequence complementarily matched with the probe sequence of an SLA-RT primer was specifically and most efficiently reverse-transcribed, and its relative abundance was subsequently determined by both end-point PCR and qRT-PCR. The accumulation of a cleaved 5′-mRNA fragment at a specific cleavage site within and near the predicted let-7:*TUSC2* target pairing sequences was represented by the relative intensity of each specific SLA–RT-PCR amplicon resolved on an agarose gel (Fig. 1a-I, upper panel) and by the relative fragment abundance (RFA) on a qRT-PCR histogram (Fig. 1a-I, lower panel).

Three blocks of intensified amplicons were detected at bases U3-C9, U15-C18 and U25-A29, corresponding to the 3′-adjacent region, the seed region, and both the 3′-supplementary pairing and 5′-adjacent regions, respectively. These SLA–RT-PCR products represented the dynamic activities mediated by let-7 on the *TUSC2* mRNA target site. The intensified amplicons that dominated at the 3′-supplementary pairing region and continued to the adjacent region, toward the 5′ end of the mRNA, with a characteristic gradually descending tail, indicated that the initially cleaved mRNA fragments at this region were at least partially subjected to the RNA 3′-5′ decay pathway. In contrast, the lack of mRNA fragment accumulation at the base block C19-U23, at the immediate 5′ side of the intensive PCR amplicon block at U15-C18, suggested that the cleaved 5′-mRNA fragments within this region were degraded by an RNA 5′-3′ decay pathway. It should be noted that the SLA-RT and PCR primers were designed to detect only the 5′-RNA fragments generated by endonucleases or 3′-5′ exonuclease activities.

### 3′-Uridylation of 5′-Fragments of Cleaved mRNA

Non-templated oligouridines with various lengths (U-tracks) could be posttranscriptionally added to the 3′ ends of miRNA-cleaved 5′-mRNA fragments in various organisms^18^. To test whether oligouridines were added to the cleaved 5′ fragments that accumulated around the let-7 target site on *TUSC2* mRNA, we modified the SLA-RT primers by adding varied numbers of adenosines at the 5′ end of the probe sequences to match the non-templated oligouridine that could be added to the 3′ ends of cleaved mRNA fragments (Supplementary Fig. 1b). These 3′-oligouridylated mRNA fragments create complementary gaps between SLA-RT primer probe sequences and their targeted 3′ terminal sequences on these cleaved mRNA fragments but perfectly match with one of the U-track–detecting SLA-RT primers with enhanced thermostability through base stacking effects. The U-track–specific SLA-RT primers were annotated as nU-SLA-RT primers, with the n representing the number of extra adenosines incorporated into the primer. SLA-RT reactions were performed with both unmodified SLA-RT and 2U-SLA-RT primers (Supplementary Table 1b) using total RNAs isolated from H1299 cells (Fig. 1a-I,II).

Amplicon intensities at the block positions U3-C8, U15-C18, U25-C27 and G28-C32 along the let-7:*TUSC2* target site (Fig. 1b-II) were significantly higher in oligouridylated mRNA fragments, as detected by 2U-SLA–RT-PCR, than those at the corresponding positions in unmodified mRNA fragments, as detected by unmodified SLA–RT-PCR reactions (Fig. 1a-I). The results from SLA–RT-PCR reactions with 8U-SLA-RT primers showed elevated amplicon intensities at these sites as well (data not shown). The RT-priming preference of nU-SLA-RT primers for the oligouridylated RNAs was further demonstrated and up to 15 nt of posttranscriptionally added oligouridines were detectable in our studies (Supplementary Fig. 2). U-tracks up to 24 nt were reported by others^18^. That the levels of mRNA fragments with varied lengths of oligouridines were greater than those of the unmodified RNA fragments suggests that the 3′-oligouridine–modified mRNA fragments are temporarily accumulated rather than degraded immediately.

To predict the actual let-7–targeted cleavage sites and their possible origins, we performed in-depth analysis of the unique pattern and relative quantity of these accumulated SLA–RT-PCR products derived from let-7–mediated *TUSC2* mRNA cleavage and 3′-uridylation activities at various positions in the let-7:*TUSC2* target site and its 5′- and 3′-adjacent regions (Fig. 1). The let-7:*TUSC2* mRNA target interaction analysis predicted strong and extended base-pairing in the seed region (Fig. 1c). The uridine base-paired G17, which was positioned at the centre of the let-7 RNA sequences near the 3′-end of the seed region, displayed a substantial accumulation of both the unmodified (Fig. 1a-I) and 3′-uridylated (Fig. 1a-II) mRNA fragments and was immediately followed by 3′-uridylation at its 3′ side close to the central bulge region, making G17 the most likely cleavage site. The U-paired G17 at the end of the miRNA seed pairing region may make it susceptible to endonucleolytic cleavage and concurrent 3′-uridylation, as observed at other potential cleavage sites with a similar location and fragment accumulation pattern, e.g., the accumulated mRNA fragments at C12 in a predicted let-7e seed region (Fig. 1a,c). The highly accumulated amplicons at U16 could result either from direct cleavage at U16 or the posttranscriptional addition of one uridine to the G17 fragment. In theory, cleavage at any of the nt in U14-U16 might produce RNA fragments identical to G17 fragments with 1–3 uridines added at their 3′-termini. The two canonically base-paired nt A26 and C27 at the predicted 3′ supplementary pairing regions displayed significant accumulation of amplicons in both unmodified and oligouridylated forms but with no apparent difference in intensity, which might represent putative cleavage sites.

### Specificity of Cleavage and Oligouridylation

To demonstrate whether the observed accumulation of cleaved and 3′-uridylated *TUSC2* mRNA fragments was specific to let-7 miRNA activity, we examined the effects of knocking down expression of endogenous let-7 miRNAs by a let-7–specific miRNA inhibitor on SLA– and U-SLA–RT-PCR outputs in H1299 cells. H1299 cells were transfected with a miR-98 locked nucleic acid inhibitor (miR-98-LNAi) that is effective in all let-7 miRNA members. The expression of let-7 miRNAs was depleted in the cells transfected with miR-98 LNAi 48 h after treatment, as shown by qRT-PCR analysis using let-7–specific SL-RT primers (Fig. 1b). The accumulation of cleaved *TUSC2* 5′-mRNA fragments was markedly reduced by this knockdown of endogenous let-7 expression (Fig. 1a-III,b, +miR-98 LNAi). The specificities of the miR-98–mediated *TUSC2* mRNA cleavage and sequential 3′-oligouridylation were further demonstrated by the dramatically different patterns and intensities of 2U-SLA–RT-PCR amplicons detected at various positions around the let-7:*TUSC2* mRNA target site between the miR-98-LNAi–treated (Fig. 1a-IV) and –untreated (Fig. 1a-II) H1299 cells. However, the accumulation of oligouridylated mRNA fragments at base positions G17, A26 and C27 remained strong 48 h after let-7 knockdown (Fig. 1a-IV), suggesting that degradation of the 3′-oligouridylated 5′-mRNA fragments was delayed.

The characteristic pattern and intensity of mRNA fragment accumulation detected surrounding the endogenous let-7:*TUSC2* target site reflected the collective activities of all let-7 family members in H1299 cells. Let-7 miRNAs differ from each other in a few bases, primarily in the central bulge region (hence the lack of base-pairing) and the 3′ supplementary pairing region, and they share a conserved short stretch of base-paired (7–10 nt) sequences at the seed region (Fig. 1c). The miRNA:target interaction algorithm used in the miRmate program^17^ allowed us to predict strong 3′ supplementary pairings that could compensate or enhance base-pairing in the miRNA seed region. With a maximum bulge loop of 3 nt permitted on the target sequence, a putative let-7 miRNA:target base-pairing map was composed according to the minimal free energy (MFE) of each pair, as calculated with the assistance of RNA Hybrid^19^, to compare the contributions from each let-7 family member to the detected target mRNA cleavage activities (Fig. 1c). As expected, the let-7–mediated *TUSC2* mRNA cleavage activities were concentrated in the intensively base-paired seed region and in the predicted supplementary base-pairing region (Fig. 1a), while no apparent let-7–mediated mRNA cleavage activities were detected in the central bulge region, which lacks miRNA:mRNA base-pairings and has a high MFE microenvironment (Fig. 1a-I,III). However, weak basal levels of SLA–RT-PCR amplicons at the base block G20-U23 in the central bulge region were always detectable in all RNA samples tested (Fig. 1a-I,III). These weak amplicon levels might represent the steady-state of 3′-5′ decayed fragments of *TUSC2* mRNAs in these cells. Undetectable oligouridylated mRNA fragments in the central bulge region confirmed the lack of let-7–mediated mRNA cleavage activity in that region (Fig. 1a-II,IV). The accumulated fragments at C18 might also be the result of similar cleavage patterns at central pairs contributed by let-7a, -7b, -7c and -7d activities (Fig. 1c). These results suggest that miRNA-mediated target mRNA cleavage requires an appropriate miRNA-mRNA base-pairing. All the potential let-7f-cleaved and 3′-uridylated fragments on target TUSC2 mRNA detected by SLA-AT-PCR were further verified and confirmed by automated DNA sequencing of the direct SLA-RT- PCR end products or their plasmid clones (Supplementary Fig. 2c).

**Figure 2:**
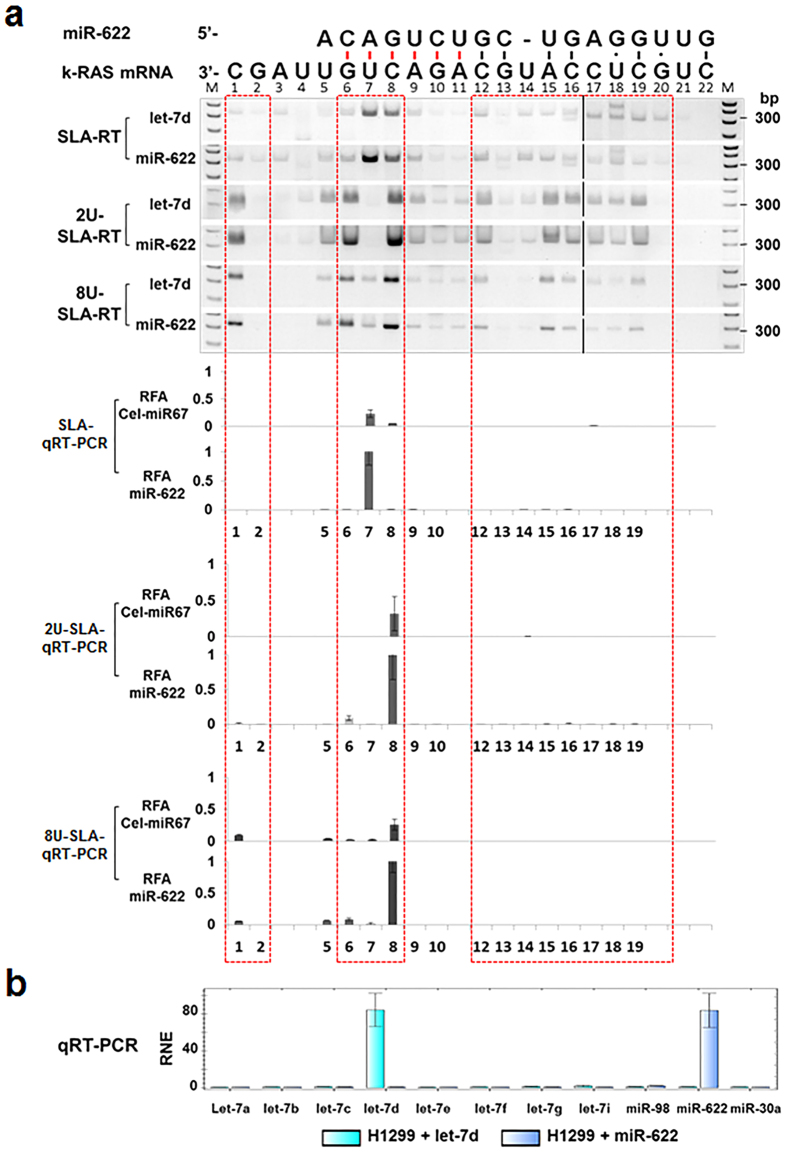
Accumulation of Cleaved Target *kRAS* mRNA 5′-fragments by miR-622 Overexpression in H1299 Cells. (**a**) *miR-622*–targeted *kRAS* mRNA cleavage was detected by SLA–RT-PCR. Total RNAs were isolated from H1299 cells transfected with a *miR-622* expression vector or a *let-7d* expression vector as a nonspecific control. RT reactions were performed using SLA-RT and 2U-SLA-RT primers (Supplementary Table 1d) for detection of cleaved *kRAS* target mRNA fragments (as indicated by red bars between the matched bases in seed region with and without 3′-uridylation, respectively, SLA–RT-PCR products were analysed by 2% agarose gel electrophoresis and shown in upper panels. M: 0.03 μg of 1 kb DNA ladder. Results of the quantitative real-time RT-PCR (SLA-qRT-PCR) analysis of *miR-622*-mediated target *kRAS* mRNA cleavage and uridylation activities at corresponding sites to the above SLA-RT-PCR reactions were represented as relative fragment abundance (RFA) compared to the highest amplicon abundance in individual qRT-PCR series and shown in lower graph panels. The mature cel-miR-67 expressing plasmid was used as a negative control. RFA values were represented as the mean of three independent experiments and error bars as standard errors to the mean. Overexpression of *miR-622* resulted in an accumulation of cleaved *kRAS* mRNA fragments at bases G6, U7 and C8, and of the 3′-oligouridylated fragments at bases G6 and C8 corresponding to the *miR-62*2 seed region but with ignorable cleavage and uridylation activities at bases C12-C20 in central bulge and supplementary base pair regions, as indicated by red boxes. (**b**) Relative normalised expression (RNE) levels of *let-7* miRNAs, *miR-622* and *miR-30a* were determined in H1299 cells transfected with either let-7d or *miR-622* expression vector by qRT-PCR.

To further define miRNA-mediated target mRNA cleavage and uridylation at specific cleavage sites on the target mRNA, we analysed the SLA–RT-PCR or SLA–qRT-PCR amplicon patterns and intensities of the cleaved and 3′-uridylated fragments of human *kRAS* mRNA directly targeted by a single miRNA species, hsa-miR-622^20^, in H1299 cells by overexpression of the *miR-622* gene, using the overexpressed *let-7d* as a nonspecific control, which was confirmed to have no predicted binding activity in this miR-622-targeted *KRAS* mRNA region (Fig. 2a). For the SLA-qRT-PCR assay, H1299 cells transfected with a mature *cel-miR-67* expressing plasmid (UCACAACCUCCUAGAAAGAGUAGA, MIMAT0000039) were used as a negative control. The *cel-miR-67* was confirmed to have minimal sequence identity with miRNAs in human, mouse, and rat and shown to have no significant biological impact when ectopically expressed in those mammalian cells. Overexpressed *miR-622* and *let-7d* mRNAs were detected by qRT-PCR in H1299 cells transfected with a *miR-622* or a *let-7* expression vector, respectively (Fig. 2b). Overexpression of miR-622 in H1299 cells resulted in increased accumulation of both the cleaved *kRAS* mRNA fragments at bases G6, U7 and C8, as detected by both SLA–RT-PCR and SLA-qRT-PCR (Fig. 2a, SLA-RT and SLA-qRT-PCR), and the 3′-oligouridylated fragments at bases G6 and C8, as detected by 2U-SLA–RT-PCR and 2U-SLA-qRT-PCR (Fig. 2a, 2U-SLA-RT and 2U-SLA-qRT-PCR), compared to their steady expression backgrounds detected in the control H1299 cells overexpressing let-7d. The markedly increased accumulation of the cleaved *kRAS* fragments at U7 in conjunction with the uridylated fragments at U7 could have been due to either miR-622–mediated direct cleavage activity at U7 or the addition of one uridine to the 3′ ends of cleaved *kRAS* fragments at C8. Increased accumulation of the 3′-uridylated fragments at C8 was detected by both 2U-SLA–RT-PCR and 2U/8U-SLA-qRT-PCR (Fig. 2a, 2U-SLA-RT, 2U-SLA-qRT-PCR, and 8U-SLA-qRT-PCR), suggesting that the miR-622–mediated cleavage of *kRAS* mRNA occurred at C8 with one uridine addition to the 3′-end of the cleaved fragment. These results, together with the patterns of accumulated fragments at the C6 and C8 positions of the *TUSC2* mRNA let-7 target site (Fig. 1a-I,II), suggest that the miRNA-mediated target mRNA cleavage occurred at specific positions on mRNA sequences and the concurrent 3′-oligouridyl modification of cleaved mRNA fragments with one or two uridyl residues may be sufficient to promote targeted 5′-3′ mRNA decay^21^,^22^.

**Figure 3:**
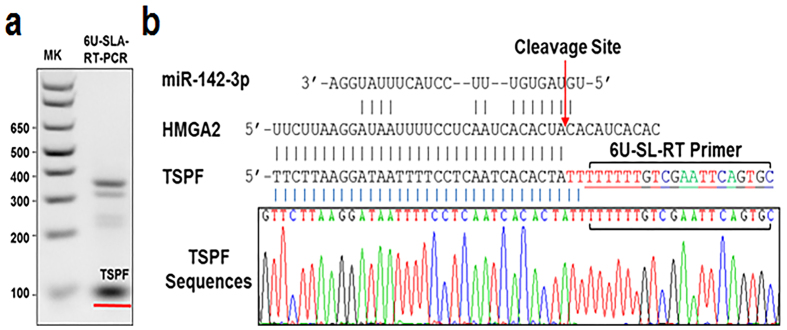
Verification of 3′-oligouridine Addition in Cleaved *HMGA2* mRNA Fragments Targeted by miR-142-3p. (**a**) 3′-oligouridine additions on 5′-fragments of cleaved *HMGA2* mRNA by miR-142-3p activity in HeLa cells by U-track–specific SLA–RT-PCR were detected by agarose gel electrophoresis. An SL-RT primer with 5′-AAAAAA-3′ (6U-SL-RT) as probe was used as the RT primer to randomly prime cleaved mRNA fragments in oligouridylated forms. The universal PCR primer and a 5′-PCR primer specific to *HMGA2* mRNA were used to amplify possible 3′-oligouridine–modified fragments, and several 6U-SLA–RT-PCR amplicons, termed oligo-thymine-specific PCR fragments (TSPFs), were detected on the agarose gel. MK: 0.03 μg of 1 kb DNA ladder. (**b**) 3′-oligouridine addition was verified by DNA sequencing. The 100-bp TSPF was dissected from the agarose gel and analysed by automated DNA sequencing.

The miRNA-mediated 3′-oligouridine addition with the exact numbers of uridine bases to the cleaved target mRNA fragments was further confirmed at the 3′-ends of specific 5′-fragments in the cleaved *HMGA2* (high-mobility group AT-hook 2) mRNA targeted by miR-142-3p activity in HeLa cells by a U-track–specific SLA–RT-PCR and Sanger sequencing (Fig. 3). A 6U-SL-RT primer was used to randomly prime cleaved *HMGA2* mRNA fragments in oligouridylated forms. All potential 6U-SLA–RT-PCR amplicons, termed oligo-thymine–specific PCR fragments (TSPFs), were detected by agarose gel electrophoresis (Fig. 3a), and individual TSPFs were then sequenced (Fig. 3b). Eight uridine residues were detected on the 3′ termini of the *HMGA2* mRNA fragment, as confirmed by sequence alignment to the seed region of the hsa-miR-142-3p target site on the *HMGA2* mRNA, which was revealed by deep sequencing data available in miRbase databases. The two additional uridine residues detected at the front of the 6U-SL-RT primer sequence clearly indicated that the detected 3′-oligouridylated fragments originated from and were posttranscriptionally added to the miR-142-3p–cleaved *HMGA2* mRNA fragments in H1299 cells and not artificially introduced by nU-SLA-RT primers and subsequent PCR amplification.

### mRNA Structural Context and miRNA Activity

**Figure 4:**
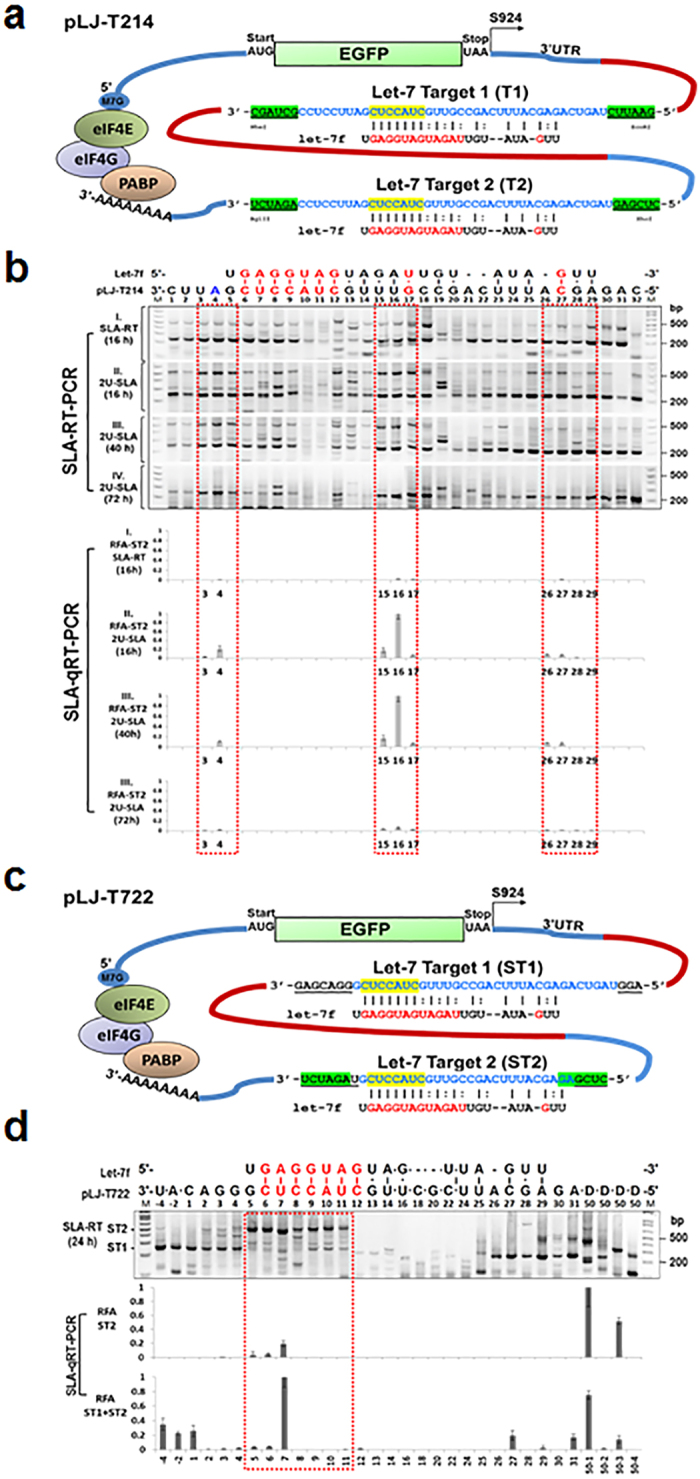
Effects of Target mRNA Structural and Positional Elements on Activities of miRNA-mediated Cleavage and Uridylation. (**a**) The plasmid construct of pLJ-T214 containing the *EGFP* reporter gene with two copies of predicted let-7a target sequences in its 3′UTR. (**b**) Cleaved mRNA 5′-fragments from let-7 targets T1 and T2 were detected by SLA–RT-PCR (upper gel panels) and SLA-qRT-PCR (lower panels). The predicted sizes of SLA–RT-PCR products derived from target sites T1 and T2 were 240-209 bp and 532-500 bp, respectively. Total RNAs were prepared from H1299 at 16, 40 and 72 h after pLJ-T214 transfection and RT reactions were performed using SLA-RT or 2U-SLA-RT primers. The corresponding target T2 cleavage and uridylation activities detected by SLA-qRT-PCR with T2 fragment specific PCR primers and TaqMan probe. (**c**) The plasmid construct of pLJ-T722. The 3′UTR of the *EGFP* reporter gene containing two copies of identical predicted let-7:target pairing sequences but with varied lengths and compositions of nt (underlined) at their 5′- and 3′-adjacent regions (**ST1** and **ST2**). (**d**) The effect of structural composition of target mRNA on let-7 miRNA–mediated mRNA cleavage activity as detected by SLA–RT-PCR assay (upper gel panels) and SLA-qRT-PCR (lower panels). Total RNAs were prepared from H1299 cells transfected with pLJ-T722 at 24 h, and SLA–RT-PCR was performed using SLA-RT primers. D50, a PCR primer specific to the pLJ-T722 transcript, was designed to bond at every 50 bases along the target sequences toward its 5′ end. The base numbers correspond to those shown in (**b**), and escaped bases are indicated by “•” between them. M: 0.03 μg of 1 kb DNA ladder. A Taqman probe-based qPCR assay was used to detect cleavage activity specific to target **ST2** and a SYBR Green-based qPCR to detect both target **ST1** and **ST2** cleavage activities, respectively. All qPCR results were presented as relative fragment abundance (RFA). Each RFA value was represented as the mean of three independent experiments and error bars as standard errors to the mean. The target cleavage and uridylation activities in miRNA target regions were highlighted in red boxes.

To determine the potential effects of structural and spatial contexts of the target mRNA on miRNA-mediated mRNA cleavage, we developed an enhanced green fluorescence protein (EGFP) reporter plasmid-based model system with a defined let-7 target and SLA–RT-PCR primer binding sequences and a fully functional mammalian mRNA structure to monitor the precise action of miRNA on its target in a time- and space-dependent manner. The reporter plasmid (pLJ-T214) consisted of an *EGFP* reporter coding sequence under the control of a cytomegalovirus (*CMV*) promoter, immediately followed by a 3′UTR with two identical copies of let-7 target sequences (T1 and T2) directly derived from *TUSC2* mRNA sequences arranged in tandem and a BGH poly(A) signalling sequence (Fig. 4a).

In pLJ-T214–transfected H1299 cells, three identical copies of *TUSC2* let-7 target sites were produced: one copy in the 3′UTR of the endogenous *TUSC2* mRNA transcript and two copies in the 3′UTR of the exogenously expressed pLJ-T214 *EGFP* reporter transcript. Endogenous let-7–mediated mRNA cleavage of those target sites would produce 5′-mRNA fragments with identical 3′ termini, which could be detected competitively by the same SL-RT primer in RT. The origins of the cleaved mRNA fragments could be determined by subsequent PCR amplification with either the endogenous or the pLJ-T214 transcript–specific PCR primers, which would generate SLA–RT-PCR amplicons of different sizes. The predicted sizes of the SLA–RT-PCR products derived from target sites T1 and T2 were 240-209 bp and 532-500 bp, respectively, and those derived from the endogenous target site were predicted to be 230-198 bp.

Cleavage activities on the T1 and T2 sites of the pLJ-T214 transcripts served as a control for verifying the endogenous *TUSC2* mRNA fragments and internal references for defining the spatial effect of target sites on endogenous let-7 activity by the SLA–RT-PCR assay or SLA-qRT-PCR. Total RNAs from pLJ-T214–transfected H1299 cells at different time points after transfection were examined with the same set of SLA-RT primers used for the endogenous *TUSC2* mRNA fragment detection (Supplementary Tables 1a and 2b). Sixteen hours after transfection, both unmodified (Fig. 4b-I) and oligouridylated (Fig. 4b-II) *TUSC2* mRNA fragments were detected along the pLJ-T214 T1 and T2 sites with predicted sizes. Although PCR conditions were optimised to minimise the size variation among T1 and T2 amplicons, the SLA–RT-PCR amplicon intensities or relative fragment abundance (RFA) by SLA-qRT-PCR in the T1 site were consistently stronger than those in its T2 counterpart at any given time; however, intensity patterns at both sites displayed similar trends over the course of transfection. The accumulation patterns of the 3′-uridylated T2 fragments at 16 h after transfection (Fig. 4b-II) and those of the 3′-uridylated T1 fragments at 72 h (Fig. 4b-IV) resembled the pattern obtained with the endogenous *TUSC2* mRNA let-7 target site (Fig. 1a-II), particularly within the base blocks defined by red boxes in Fig. 4b. The uridylated fragments from T2 were almost undetectable at 72 h after transfection. For these two identical target sites located at different positions in the same transcript, the upstream T1 appeared to have more let-7–mediated cleavage activity than the downstream T2, suggesting a potential positional advantage of T1 over T2 in accessing let-7–guided miRISCs and miRISC scanning along mRNA sequences in the 5′ to 3′ direction.

Unlike siRNAs or plant miRNAs, most mammalian miRNAs display no extended base-pairing to their targeted mRNA sequences and they target mainly at 3′UTRs, where extensive secondary and tertiary RNA structures often exist^1^,^2^,^23^. To determine the structural effect of the target mRNA on let-7 miRNA–mediated mRNA fragment distribution, we constructed a plasmid vector pLJ-T722 (Fig. 2c) similar to the pLJ-T214 plasmid (Fig. 4a), in which the two copies of identical let-7:target pairing sequences in the 3′UTR of the *eGFP* reporter gene had the length and composition of nt adjacent to their 5′- and 3′- ends altered, labelled as ST1 and ST2 (Fig. 4c). The base alterations in ST1 and ST2 could change the folding patterns and local MFE attributions of the target RNA sequences, thus altering miRNA target recognition, binding, and activity. The endogenous let-7–mediated mRNA cleavage activity on the ST1 and ST2 target sites was examined by SLA–RT-PCR or SLA-qRT-PCR (only at sites whining the red boxes), using total RNAs prepared from H1299 cells transfected by pLJ-T722 at 24 h as templates and the SLA-RT primers described in Supplementary Table 1c (Fig. 4d). The accumulated mRNA fragments cleaved from the ST2 target site at the 5′ adjacent region in pLJ-T722 transcripts were consistently visible over the 250-bp gap between the ST2 and ST1 target sites but were blocked at the 3′ front of the ST1 seed region (Fig. 4d), similar to those observed at T2 in pLJ-T214 transcripts (Fig. 4b). However, the mRNA fragment accumulation activities between ST2, ST1 in pLJ-T722 and T2, T1 in pLJ-T214 transcripts were dramatically different. These results indicate that the structural context of the target mRNA can influence miRNA-mediated target cleavage.

### mRNA Cleavage and 3′-Uridylation

**Figure 5:**
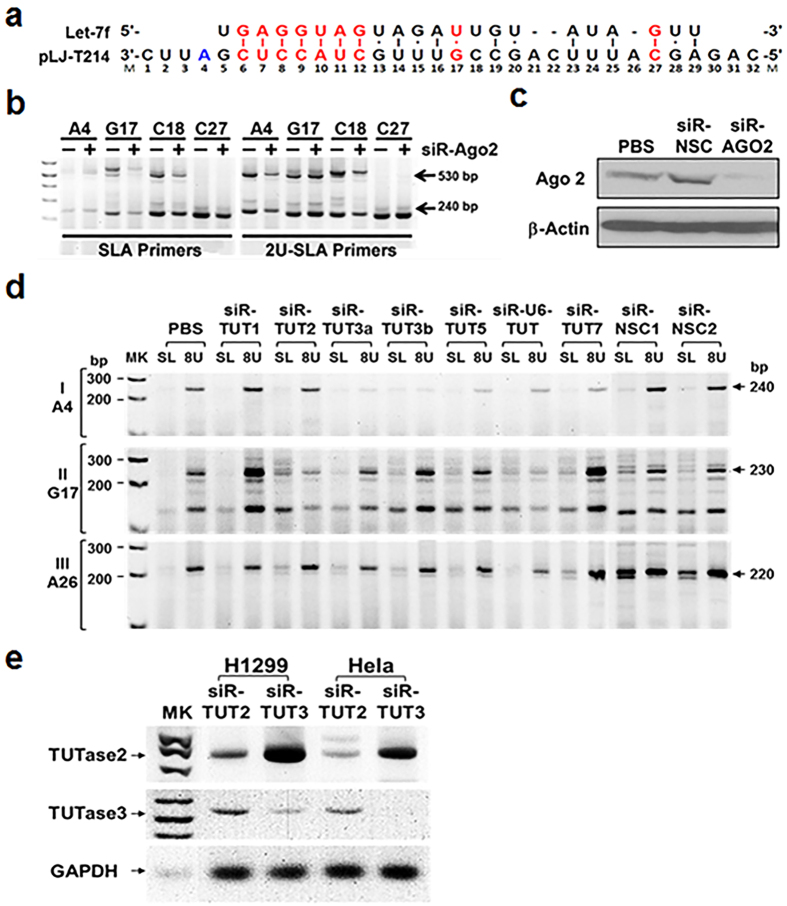
Inhibition of let-7–mediated Target mRNA Cleavage and 3′-uridylation by Ago2- and TUTase-specific siRNA Inhibitors. (**a**) The predicted let-7:target mRNA pairing sequences derived from the pLJ-T214 *EGFR* reporter transcript. (**b**) Effects of Ago2 knockdown on let-7–mediated target mRNA cleavage and uridylation. Total RNAs were prepared from H1299 cells treated with siR-Ago2, and RNAs from untreated cells and siR-NSC-treated cells were used as a negative and a nonspecific control, respectively. The unmodified and 3′-oligouridylated 5′-mRNA fragments of the pLJ-T214 transcript (see Fig. 3a) cleaved by *let-7* activity were determined by SLA–RT-PCR using selected SLA-RT and 2U-SLA-RT primers, respectively, with corresponding nt residue numbers indicated in (**a**) and described in Supplementary Table 1e. SLA–RT-PCR amplicons of 528 bp and 234 bp were expected on agarose gel for *let-7*–cleaved mRNA fragments from target sites T1 and T2, respectively. M: 0.03 μg of 1 kb DNA ladder. (**c**) Effect of siR-Ago2 on Ago2 protein expression by western blotting. PBS, phosphate buffered saline. (**d**) Effects of knockdown of *TUTase* gene expression on let-7–mediated target mRNA cleavage and uridylation. Total RNAs were prepared from H1299 cells treated with TUTase-specific siRNAs (siR-TUT1, siR-TUT2, siR-TUT3a/3b, siR-TUT5, siR-U6-TUTase, and siR-TUT7 respectively), and two scrambled siRNAs were used as nonspecific controls (siR-NSC1 and 2). The unmodified and 3′-oligouridylated 5′-mRNA fragments of the pLJ-T214 transcript cleaved by let-7 activity were determined by SLA–RT-PCR using selected SLA-RT (SL) and 8U-SLA-RT (8U) primers, respectively, with corresponding nt residue numbers (A4, G17 and A26) indicated in (**a**) and described in Supplementary Table 1e. MK: 0.03 μg of 1 kb DNA ladder. (**e**) Effect of siR-TUTs on *TUTase2* and *TUTase3* gene expression in H1299 and HeLa cells by RT-PCR.

The human Argonaute protein superfamily consists of endonucleolytic-capable Ago2 protein and noncatalytic Ago1, Ago3 and Ago4 proteins^24^,^25^. Ago2, but not other Argonaute proteins, has been implicated in a specific role in endonucleolytic processing and posttranscriptional regulation of miRNAs and in miRNA-mediated target gene silencing in miRISCs in mammalian cells^26^,^27^. To determine whether Ago2 was involved in the let-7–mediated target mRNA endonucleolytic cleavage in miRISC, we analysed the effects of Ago2 knockdown on let-7–mediated target mRNA cleavage and uridylation in H1299 cells co-transfected with the pLJ-T214 plasmid and Ago2-specific siRNA (siR-Ago2) (Fig. 5a–c). The unmodified and 3′-oligouridylated 5′-mRNA fragments of the cleaved pLJ-T214 transcript, at the selected corresponding nt residues A4, G17, C18 and C27 on target mRNA sequences (Fig. 5a), were examined by SLA–RT-PCR using SLA-RT and 2U-SLA-RT primers in H1299 cells 24 h after transfection (Fig. 5b). Ago2 protein expression was significantly reduced upon *Ago2* gene knockdown by siR-Ago2 in these H1299 cells (Fig. 5c). After Ago2 expression knockdown, the cleaved A4 mRNA fragments from the pLJ-T214 T1 site remained unchanged in the unmodified form but decreased significantly in the oligouridylated form. In contrast, G17 and C27 fragments were reduced in the unmodified form but remained the same in the oligouridylated form, whereas C18 fragments decreased in both the unmodified and oligouridylated forms (Fig. 5b). These observations suggested that the endonuclease activity of Ago2 was directly responsible for the let-7–guided target mRNA cleavage activity at G17 and C27, leading to progressive reduction of the 3′-uridylated fragments by 3′-uridylation–facilitated 5′-3′ RNA decay. The significant reduction of oligouridylated mRNA fragments at A4 and C18 suggests the indirect involvement of Ago2 in let-7–mediated target mRNA cleavage, where the majority of detected mRNA fragments at T1 originated from the 3′-5′ decay products of cleaved 5′-miRNA fragments at the T2 site.

Terminal uridylyl transferases (TUTases) catalyse the transfer of UMP residues to the 3′ hydroxyl group of RNA and are involved in bulk degradation of histone mRNA in human cells^22^,^28^,^29^. To investigate the involvement of TUTases in miRNA-mediated mRNA cleavage and 3′-uridylation of the cleaved mRNA fragments within and near the miRISC, we analysed the effects of *TUTase* gene knockdown on let-7–mediated target mRNA 3′-oligouridylation in H1299 cells treated with siRNAs specific to all known mammalian TUTases by SLA–RT-PCR (Fig. 5d,e). Total RNAs were prepared from H1299 cells co-transfected with the pLJ-T214 plasmid and TUTase–specific siRNAs, and two scrambled siRNAs were used as nonspecific controls (Fig. 5d). The unmodified and 3′-oligouridylated 5′-mRNA fragments of the pLJ-T214 transcript cleaved by let-7 activity were determined by SLA–RT-PCR using selected SLA-RT and 8U-SLA-RT primers, corresponding to nt at A4, G17 and A26 (Fig. 5a and Supplementary Table 1e). The siRNA-mediated downregulation of expression of *TUTase2* and *TUTase3* genes (Fig. 5e) showed most promising effect on inhibition of 3′-uridylation of the cleaved target mRNA fragments among all siR-TUTs tested (Fig. 5d). The 3′-uridylation activity levels at A4 in the 3′-adjacent region of the pLJ-T214 transcripts were significantly lower in H1299 cells treated with siR-TUT3 but not those treated with siR-TUT2 compared to untreated or scrambled siRNA–treated controls (Fig. 5d). In contrast, the 3′-uridylation activity levels at G17 in the seed region were significantly lower in cells treated with siR-TUT2 but not in those treated with siR-TUT3 (Fig. 5d). No apparent changes in 3′ uridylation at A26 in the central bulge region were observed after knockdown of either TUTase2 or TUTase3 (Fig. 5d). The oligouridine addition to the 3′-termini of mRNA fragments generated by miRNA-guided and Ago2-catalysed endonucleolytic cleavage at cleavage sites either in the seed regions or the 3′ supplementary pairing regions may be mediated by the activity of TUTase2, while the accumulated uridylated fragments at unpaired bases in the miRNA:mRNA target site within the miRISC may be derived from the 3′-uridylated 3′-5′ decayed RNA fragments at their downstream cleavage sites on the same mRNA. The uridylation outside the miRISC or in its proximity may be catalysed by TUTase3 or other TUTases in the cytosol.

## Discussion

Multiple models have been proposed for the molecular mechanics of miRNA-mediated posttranscriptional repression of target gene expression either by inhibition of mRNA translation, with no apparent impact on mRNA stability, or by initiation of mRNA decay^1^,^2^,^7^,^8^,^30^,^31^. Although mammalian miRNAs have been shown to decrease target mRNA levels, leading to reduced protein output^7^, direct evidence for miRNA-mediated target mRNA cleavage and degradation has rarely been obtained in mammalian cells. Our results, using a novel SLA–RT-PCR assay, show endogenous let-7 miRNA–guided and Argonaute-catalysed endonucleolytic cleavage of target *TUSC2* mRNAs at various sites in partially paired miRNA:mRNA sequences, predominantly within the miRNA seed region or in the 3′ supplementary pairing region. Most of the cleaved mRNA 5′-fragments were oligouridylated at their 3′-termini by TUTase2 in the miRISC, while some 3′-5′ decayed mRNA fragments could also be intercepted by miRISCs stationed on their targets and oligouridylated by other TUTases in the proximity of the miRISC. These 3′-oligouridylated mRNA 5′-fragments accumulated in cytosol and appeared destined for 5′-3′ decay. These findings provide direct evidence for mammalian miRNA-mediated gene silencing by target mRNA recognition, cleavage and 3′-uridylation, leading to degradation of the protein coding sequence–containing 5′-mRNA fragments via a possible bulk 5′-3′ decay pathway.

Our findings suggest that the mRNA cleavage and 3′-oligouridylation activities mediated by mammalian miRNAs with partial base-pairing to their target mRNA sequences in Ago2-miRISCs may be a common molecular mechanism for miRNA-targeted gene silencing. We have shown that target gene silencing mediated by let-7 family miRNAs and other human miRNAs could be initiated by Ago2-catalysed endonucleolytic cleavage on base-paired miRNA:mRNA target sites, which is consistent with the fact that the Ago2 endonucleolytic RNase H domain prefers paired bases as substrates^15^,^24^. Argonaute genes evolved from eukaryotic translation initiation factors and have been extensively studied, under the names eIF2C1 to eIF2C4, as translation initiators^32^. An m^7^G cap-binding motif has been identified on Argonaute MID domains but was shown to be weak and could not compete with the cap-binding eIF4E to sustain translational suppression^33^,^34^. However, this weak cap-binging motif might have provided the essential molecular basis for the initiation of miRNA-guided Ago2-miRISC scanning along the mRNA target sequences in the 5′-3′ direction. Based on the unique and dominant locations of the cleaved fragments accumulated in the seed region and the 3′ supplementary pairing region, it is possible that the miRISC stationed on miRNA:mRNA target sites could provide a barricade to the exonuclease-mediated 3′-5′ RNA decay machinery and facilitate the recruitment of specific TUTases or uridyl polymerases to the miRISC for adding oligouridines to 3′-termini of the cleaved 5′ mRNA fragments. This miRISC-imposed blockage to RNA 3′-5′ decay may also imply a cooperative role of noncatalytic miRISCs in the gene silencing pathway.

Most of the cleaved 5′ mRNA fragments were oligouridylated at their 3′-termini in the miRISC. Those 5′ mRNA fragments derived from the initial cleavage sites on the same transcript that escaped oligouridine modification in the miRISC could be subjected to the exonucleolytic RNA 3′-5′ decay pathway and would then be intercepted and oligouridylated by other TUTases in proximity to the miRISC. These oligouridylated mRNA fragments could be recognised by the cytoplasmic heptameric Lsm1-7 complex and accumulated in P-bodies^28^,^35^, which have been shown to be hubs for transient RNA species^36^,^37^,^38^,^39^,^40^. The accumulated 3′-oligouridylated mRNA fragments would be degraded through a facilitated bulk RNA 5′-3′ decay pathway within the P-bodies. The delayed action of mRNA degradation could desynchronise the lineage relationship of mRNA stability and protein output, which may partially explain the conflicting observations of miRNA-mediated gene silencing either by inhibition of mRNA translation with no apparent impact on mRNA stability or by initiation of mRNA decay^41^,^42^,^43^,^44^.

Our results provide new insights into the molecular mechanics of mammalian mRNA-mediated gene silencing by showing a cooperative action of miRNA, Ago2/TUTases and other essential molecular components in miRISCs mediating mRNA degradation initiated by mRNA cleavage and concurrent 3′-uridylation. Further studies are needed to understand the precise mechanism of miRNA action in association with specific co-factors in miRISC on posttranscriptional regulation of target gene expression in a complex mammalian cellular system.

## Methods

### Materials

RNase inhibitor was acquired from New England Bio-Labs (M0314L). DNA primers and RNA oligos were synthesized by Sigma-Aldrich. High-capacity cDNA first-strand synthesis kit, TaqMan Gene Expression Master Mix and TaqMan probes for miRNA quantification were purchased from Applied Biosystems (Life Technologies). The miRCURY LNA inhibitor against hsa-miR-98 was obtained from Exiqon. ExoSAP-IT for PCR Product Cleanup was obtained from Affymetrix. TRIzol LS Reagent, SYBR Green I and 1 Kb-Plus DNA Ladder were obtained from Invitrogen (Life Technologies). Rabbit anti-Ago2 monoclonal antibody was purchased from Cell Signaling Technology. SiGENOME SMARTpool-EIF2C2 anti-Ago2 and ON-TARGET plus non-targeting siRNA as nonspecific controls (siR-NSCs) were purchased from Thermo Scientific.

### Cell culture and transfection

Human H1299 large cell lung cancer and HeLa cervical cancer cells were grown in Roswell Park Both Memorial Institute 1640 medium and Dulbecco modified Eagle medium supplemented with 10% foetal bovine serum (FBS). For transfection, plasmid DNA was adjusted to 0.5 μg/μL and siRNAs (25 μM) were dissolved in sterile water. Plasmid DNA or siRNA was mixed with an equal volume of 8 mM DOTAP:Cholesterol liposome to make DOTAP:Chol:DNA complexes. DOTAP:Cholesterol liposomes were prepared in-house as described previously^16^. Cells were seeded at an initial density of 20–25% confluence in 6-well plates or 60-mm or 100-mm culture dishes according to experimental purposes and grown for at least 24 h before any treatment. For DNA transfection or RNAi knockdown, we added 4 μL of DOTAP:Chol:DNA complexes to 35-mm dishes with 2 mL medium containing 10% FBS, and transfection dosages were scaled according to the volume of medium in use. Cells were then returned to incubators and were mixed occasionally during transfection. After 24 h of transfection, culture media were refreshed and cells were grown until harvesting at desired time points.

### RNAi gene knockdown of Let-7 family miRNAs, Ago2 and TUTases

miRNA let-7 knockdown was carried out in H1299 cells transfected with 10 μM of miRCURY LNA anti-miR-98 inhibitor packaged in a DOTAP:Chol:siRNA complex. After 24 h of initial transfection, the medium was refreshed and cultures were returned to incubation for an additional 24 h. Cells were then subjected to direct lysis in TRIzol LS Reagent, and total RNAs were isolated according to the vendor’s recommendation. The let-7 miRNA levels were measured by qRT-PCR assays. Several putative TUTases have been identified in the human genome on the basis of a homology comparison with the U6-terminal transferase and the *Schizosaccharomyces pombe* Cid1 enzyme, which showed that TUTase1 and TUTase3 are involved in histone mRNA uridylation^28^.

To determine the effect of TUTase knockdown on miRNA-mediated fragment distribution, we synthesized all TUTase siRNAs and PCR primers according to the ref. 28. H1299 and HeLa cells plated in 6-well plates were co-transfected with 25 pmol of siRNAs and 0.1 μg of dual target reporter plasmid pLJ-T214. Twenty-four hours after co-transfection, total RNAs were isolated and *TUTase* mRNA levels were measured by end-point RT-PCR assays as reported by Mullen *et al*.^28^. The abundances of mRNA fragments with or without uridine modification were measured by SLA–RT-PCR. The siRNA and PCR primers used for monitoring TUTase knockdown and mRNA level are listed in Supplementary Table 2. The Ago2 knockdown experiments, in H1299 cells seeded in 6-well plates, were performed similarly, with co-transfection of 0.1 μg of pLJ-T214 and 25 pmol of siGENOME SMARTpool-EIF2C2 anti-Ago2 siRNA or 25 pmol of siR-NSC. Ago2 protein level was monitored by western blot assay with rabbit anti-Ago2 antibody as reported previously^16^.

### SLA–RT-PCR Assay

#### Reverse-transcription

RNAs (50 μg) were treated with 10 μL of RNase-free DNase I (M0303L, New England Biolabs) and 20 μL of RNase inhibitor in 100 μL of reaction solution for 5 min at 37 °C, 10 min at 80 °C and 15 sec at 95 °C. RT master mixes were prepared using a High-Capacity cDNA Reverse Transcription Kit (Applied Biosystems), and aliquots containing 0.25 μg RNA were mixed with 2.5 μL of the appropriate 100 nM SL-RT primers. The reactions were incubated by the following program: 1 min at 20 °C and 10 sec at 37 °C for 60 cycles, 30 min at 37 °C, 20 min at 42 °C, 5 min at 80 °C and hold at 4 °C. The RT products were then subjected to further PCR amplification for end-point PCR evaluation or to real-time PCR for quantitation. Samples not subjected to RT were used as negative controls to avoid any potential DNA contaminants in the RNA preparations.

#### PCR assay

For end-point PCR analyses of cDNA products primed by SLA-RT primers, PCR was performed in TaqMan Gene Expression PCR Master Mix with universal primer and transcript-specific primers. The RT product (1 μL) was mixed in 30 μL of PCR mixtures containing 0.5 μM PCR primers. The PCR program started at 95 °C for 10 min, followed by 30 cycles of 15 sec each at 95 °C, 1 min at 68 °C and 20 sec at 72 °C for an additional 5 cycles, chased with 15 sec at 95 °C, 2.5 min at 68 °C and 2.5 min at 72 °C. PCR products were analysed by electrophoresis on 3% agarose gels in 1× Tris-Boric acid buffer^3^.

#### qRT-PCR assays

For profiling of relative quantitative mRNA fragment abundance, a real-time SLA-qRT-PCR with ether the TagMan probe-based or SYBR-Green-based method was performed with 1 μL of RT product primed by each SLA-RT primer in triplicate in 40 μL of TaqMan Gene Expression PCR Master Mix with or without containing SYBR Green I, 0.5 μM of universal primer and 0.5 μM of transcript-specific primer. PCR reactions started at 95 °C for 10 min, followed by 10 cycles of pre-amplification of 15 sec at 95 °C, 2.5 min at 68 °C and 2.5 min at 72 °C, then 40 cycles of 15 sec at 95 °C, 1 min at 68 °C and 20 sec at 72 °C on a CFX384 Real-Time PCR Detection System (Bio-Rad). Melting curve analysis was used to confirm a single PCR product in each reaction. RNU44 was measured as a sample control, and RNA fragment relative abundance was calculated by the 2^∆Ct^ method, where normalised ∆Ct = Ct of Sample − Ct of NTC (non-template control). The qRT-PCR conditions for miRNA detection were modified to 10 min at 95 °C, 5 cycles of pre-amplification of 15 sec each at 95 °C, 2.5 min at 61 °C and 2.5 min at 72 °C, then 40 cycles of 15 sec at 95 °C, 1 min at 60 °C and 1 sec at 72 °C. Let-7 family miRNA, hsa-miR-622, hsa-miR-30a and hsa-RNU44 in H1299 cells were determined by SLA–RT-PCR methods. The data were first normalised against hsa-RNU44, and then the changes in miRNA expression level were calculated by the following formula: ∆∆Ct = ∆Ct of miRNA target − ∆Ct of miRNA negative control^45^,^46^,^47^. SL-RT primers for each miRNA are listed below in Supplementary Table 3. The qPCR primers used in each figures were described in Supplementary Information. The SLA-RT-PCR technique was described in details somewhere else^14^.

### Plasmid Construction

#### pLJ-T214 let-7 dual-target expression vector

A DNA fragment with two copies of let-7 targets identical to the *TUSC2* let-7 target site was synthesized by GenScript. A DNA insert was released by *Bsr*GI/*Bgl*II and cloned into an *EGFP* expression cassette as 3′UTR under a *CMV* promoter. The transcript 3′UTR region on the plasmid was verified by Sanger sequencing. The details of the pLJ-T214 transcript are illustrated in Fig. 3a. The first let-7 target site from the 5′ cap was annotated as T1 (2nd underlined section) and the second let-7 target as T2 (3rd underlined section). The reporter transcript of pLJ-T214 is illustrated in a circularised form with eIF4E, eIF4G and PABP bringing together the m7G-caped 5′-head and the poly(A) tail of the mRNA transcript, as proposed by Wells *et al*.^47^,^48^.

*EGFP* open reading frame        stop codon

**CGGCATGGACGAGC*TGTACA*AGTA**CCA**TAA**CCTTCAGCTGTGAGCGAAGAAACTCCCAGGCTCAATCAAGGTGTGGCTTCCATTGAGGAGCCCAGGCTGCCACAACCCTGAATAAACTCTGTTGGCGGTCGGTCCACAGTATTGGTTGGTGTTGGTTTGTGTGTGGACAAGAATTCTAGTCAG**AGCATTTCAGCCGTTTGCTACCTCG**ATTCCTCCGCTAGCTAAAGGACTGACCGGCAAGTTGGACGCCCGCAAGATCCCCATAACCTTCAGCTGTGAGCGAAGAAACTCCCAGGCTCAATCAAGGTGTGGCTTCCATTGAGGAGCCCAGGCTGCCACAACCCTGAATAAACTCTGTTGGCGGTCGGTCCACAGTATTGGTTGGTGTTGGTTTGTGTGTGGACAAGAGGTAGTCAGAGCCTGATTCTTCCGACCATTTGTTCCCGCCTTCAGATGCTCGAGCTAGTCAG**AGCATTTCAGCCGTTTGCTACCTCG**ATTCCTCC*AGATCT*GATAAACCCGCTGATCAGCCTCGA-BGH poly(A) Signal.

#### pLJ-T722 let-7 dual-target expression vector

A DNA fragment with two copies of let-7 targets identical to the *TUSC2* let-7 target site was synthesized by GenScript. A DNA insert was released by *Bsr*GI/*Bgl*II and cloned into an *eGFP* expression cassette as 3′UTR under a *CMV* promoter. The transcript 3′UTR region on the plasmid was verified by Sanger sequencing. The details of the pLJ-T722 transcript are illustrated in Fig. 3c. The first let-7 target site from the 5′ cap was annotated as T1 (2nd underlined section) and the second let-7 target as T2 (3rd underlined section).

*EGFP* open reading frame        stop codon

**CGGCATGGACGAGC*TGTACA*AGTA**AAGCGGCCGCCATAACCTTCAGCTGTGAGCGAAGAAACTCCCAGGCTCAATCAAGGTGTGGCTTCCATTGAGGAGCCCAGGCTGCCACAACCCTGAATAAACTCTGTTGGCGGTCGGTCCACAGTATTGGTTGGTGTTGGTTTGTGTGTGGACAAGAGGTAGTCAG**AGCATTTCAGCCGTTTGCTACCTCG**GGACGAGGTGCCTAAAGGACTGACCGGCAAGTTGGACGCCCGCAAGATCCCCATAACCTTCAGCTGTGAGCGAAGAAACTCCCAGGCTCAATCAAGGTGTGGCTTCCATTGAGGAGCCCAGGCTGCCACAACCCTGAATAAACTCTGTTGGCGGTCGGTCCACAGTATTGGTTGGTGTTGGTTTGTGTGTGGACAAGAGGTAGTCAGAGCCTGATTCTTCCGACCTACCTGCCATTTGTTCCCGCCTTCAGATGCTCGAG**AGCATTTCAGCCGTTTGCTACCTCG**T*AGATCT*GATAAACCCGCTGATCAGCCTCGA-BGH poly(A) Signal.

#### pLJ-T241:miR-622 expression vector

PCR was performed on genomic DNA extracted from normal human bronchial epithelial cells with the following primers flanking the miR-622 precursor:

GGACTAGTGAATTCTTTAGAGAAGCTGGACAAGTACT

ACTTGTAACTCGAGAAAAGTGTCATCTCAGCAGCTC

PCR fragments were digested with *Spe*I/*Eco*RI and cloned into a *CMV* promoter–driven expression plasmid with BGH poly(A) signal. The plasmid expression cassette was verified by Sanger sequencing.

#### pLJ-T243:let-7d expression vector

The following oligonucleotides were synthesized by Sigma-Aldrich and annealed as inserts. A *CMV* promoter–driven expression plasmid with BGH poly(A) signal was digested by *Eco*RI/*Xho*I and purified as a cloning vector. The correct clones were screened and verified by Sanger sequencing as a let-7d expression vector.

AATTCCCTAGGAAGAGGTAGTAGGTTGCATAGTTTTAGGGCAGGGATTTTGCCCACAAGGAGGTAACTATACGACCTGCTGCCTTTCTTAGGC

TCGAGCCTAAGAAAGGCAGCAGGTCGTATAGTTACCTCCTTGTGGGCAAAATCCCTGCCCTAAAACTATGCAACCTACTACCTCTTCCTAGGG

### SLA-RT-PCR Primer Design and Cleaved RNA Fragment and Oligouridine Detection

SLA-RT primer was composed of stem loop and probe as reported by Chen *et al*.^15^. The basic structures of SLA-RT primers and oligouridine-detecting 2U-SLA-RT primers are illustrated in Supplementary Fig. 1a,b. When hybridised with RNA fragments with a matching 3′-terminal, the priming capability of the SL-RT primer was enhanced through the base stacking effect^15^. The design and sequences of SLA–RT-PCR primers for miRNA detection, let-7–cleaved *TUSC2* 5′-mRNA fragment detection and quantification and nU-track detection, and the TUTase-siRNAs for each experiment, are summarised in Supplementary Tables 1a–g, 2, and 3, respectively.

### Priming Capabilities of Oligouridine-detecting nU-SLA-RT Primers

RNA fragment abundance varies with oligouridine lengths (see Supplementary Fig. 2). Let-7–mediated H1299 *TUSC2* mRNA fragments with various uridine tails were detected by qRT-PCR assay. The A4 RNA fragments of pLJ-T214 transcripts were represented by 528-bp and 234-bp amplicons and detectable in both unmodified and oligouridylated forms in total RNA samples from H1299 cells transfected with plasmid pLJ-T214. The A4 RNA fragments were further tested with SL-RT primers with different probes to confirm the oligouridine detection methods. Total RNAs from pLJ-T214-transfected H1299 cells were reverse-transcribed with 5 SL-RT primers with different probe compositions to compare their priming capabilities, and the results are shown in Supplementary Fig. 3. The SL-RT primer detected low levels of amplicons (Lane 1), as expected, but the 2U-SLA-RT primer detected elevated levels of amplicons (Lane 2). Lanes 1 and 2 were used as baselines for A4 RNA fragments in unmodified and 2U-added forms, respectively. The probe of primer 3 was separated by 2 deoxycytidines for comparison, and this change eliminated PCR amplicons completely (Lane 3). Replacing AA of primer 2 with AT or TA for primers 4 and 5 reduced the amplicon intensities (Lanes 4 and 5). The reduced A4 intensities detected with primers 4 and 5 confirmed previous reports^18^,^40^ that adenosines might be casually inserted into oligouridine tracks.

## Additional Information

**How to cite this article**: Xu, K. *et al*. MicroRNA-mediated target mRNA cleavage and 3′-uridylation in human cells. *Sci. Rep.* **6**, 30242; doi: 10.1038/srep30242 (2016).

## Supplementary Material

Supplementary Information
