# Supplementary material for: MicroRNA-mediated target mRNA cleavage and 3′-uridylation in human cells
Source: Sci Rep. 2016 Jul 21;6:30242. doi: 10.1038/srep30242 (PMC4954961; doi:10.1038/srep30242)
Supplement: Supplementary Information [file srep30242-s1.pdf]

Supplemental information

MicroRNA-mediated target mRNA cleavage and 3'-uridylation in human cells

Kai Xu, Jing Lin, Roza Zandi, Jack A. Roth, and Lin Ji

Supplemental Figures

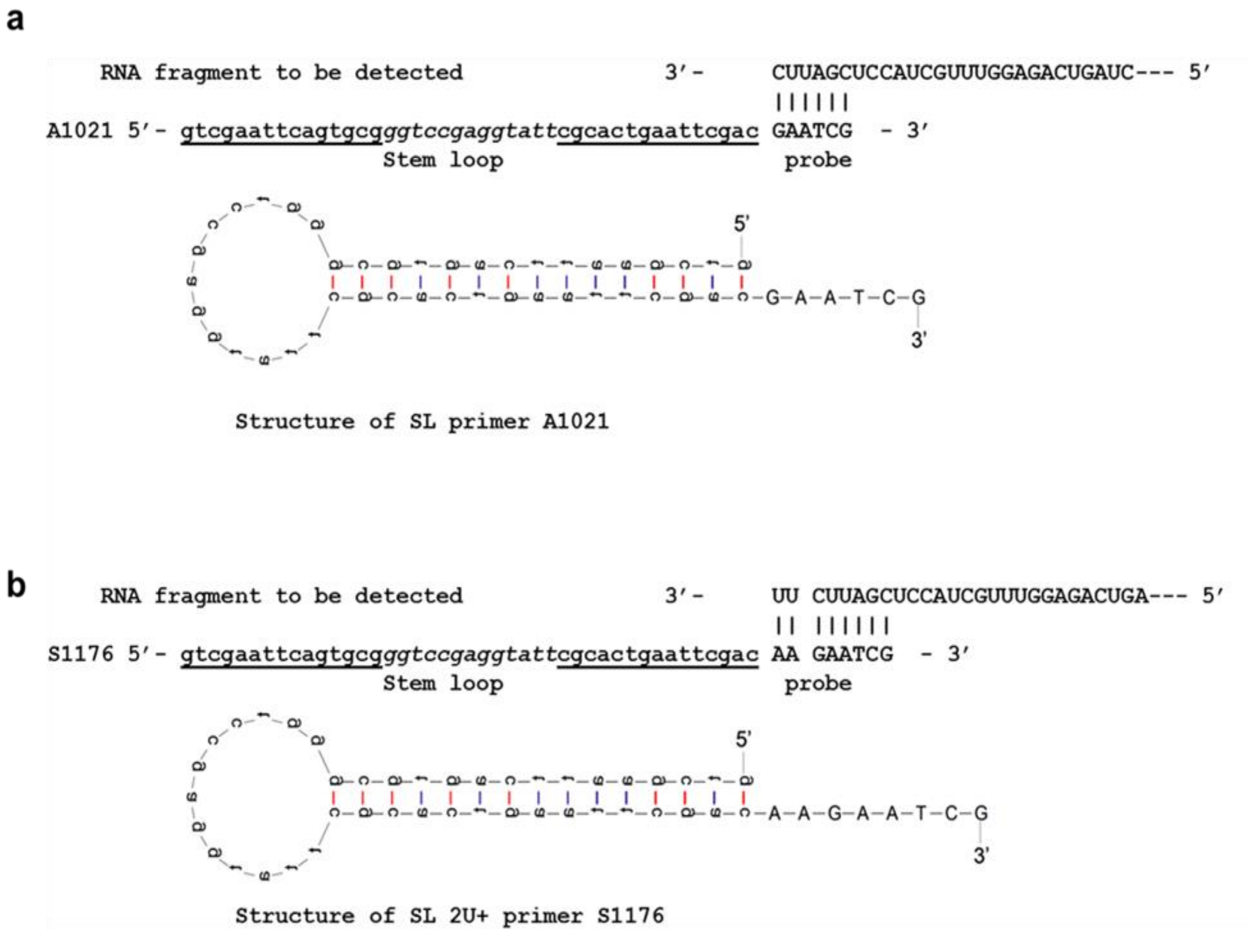

Supplementary Figure 1. Structures of the SLA-RT Primer and U-track–specific SLA-RT Primers.
(**a**) The structure of the SLA-RT primer with a 6-nt probe and its predicted RNA sequences at 3'-termini of the cleaved target 5'-mRNA fragment.
(**b**) The structure of 2U-SLA-RT primer with 2 AA plus a 6-nt probe for detection of 3'-uridylated mRNA fragment.

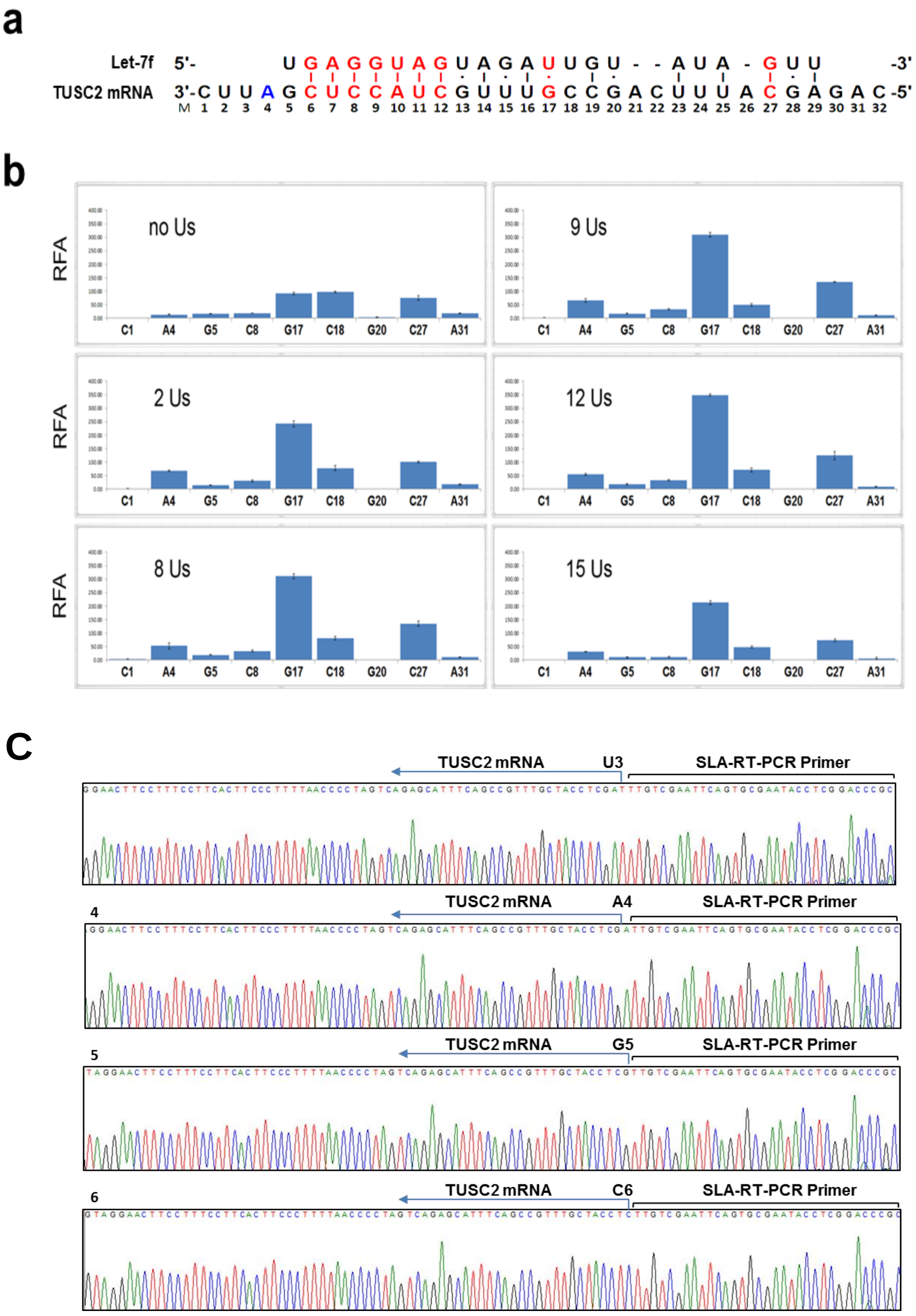
a
Let-7f 5'- U G A G G U A G U A G A U U G U - - A U A - G U U -3'
TUSC2 mRNA 3'-C U U A G C U C C A U C G U U U G C C G A C U U U A C G A G A C -5'
M 1 2 3 4 5 6 7 8 9 10 11 12 13 14 15 16 17 18 19 20 21 22 23 24 25 26 27 28 29 30 31 32
b
RFA
no Us
2 Us
8 Us
9 Us
12 Us
15 Us
C1 A4 G5 C8 G17 C18 G20 C27 A31
C
TUSC2 mRNA
U3
SLA-RT-PCR Primer
4
A4
5
G5
6
C6

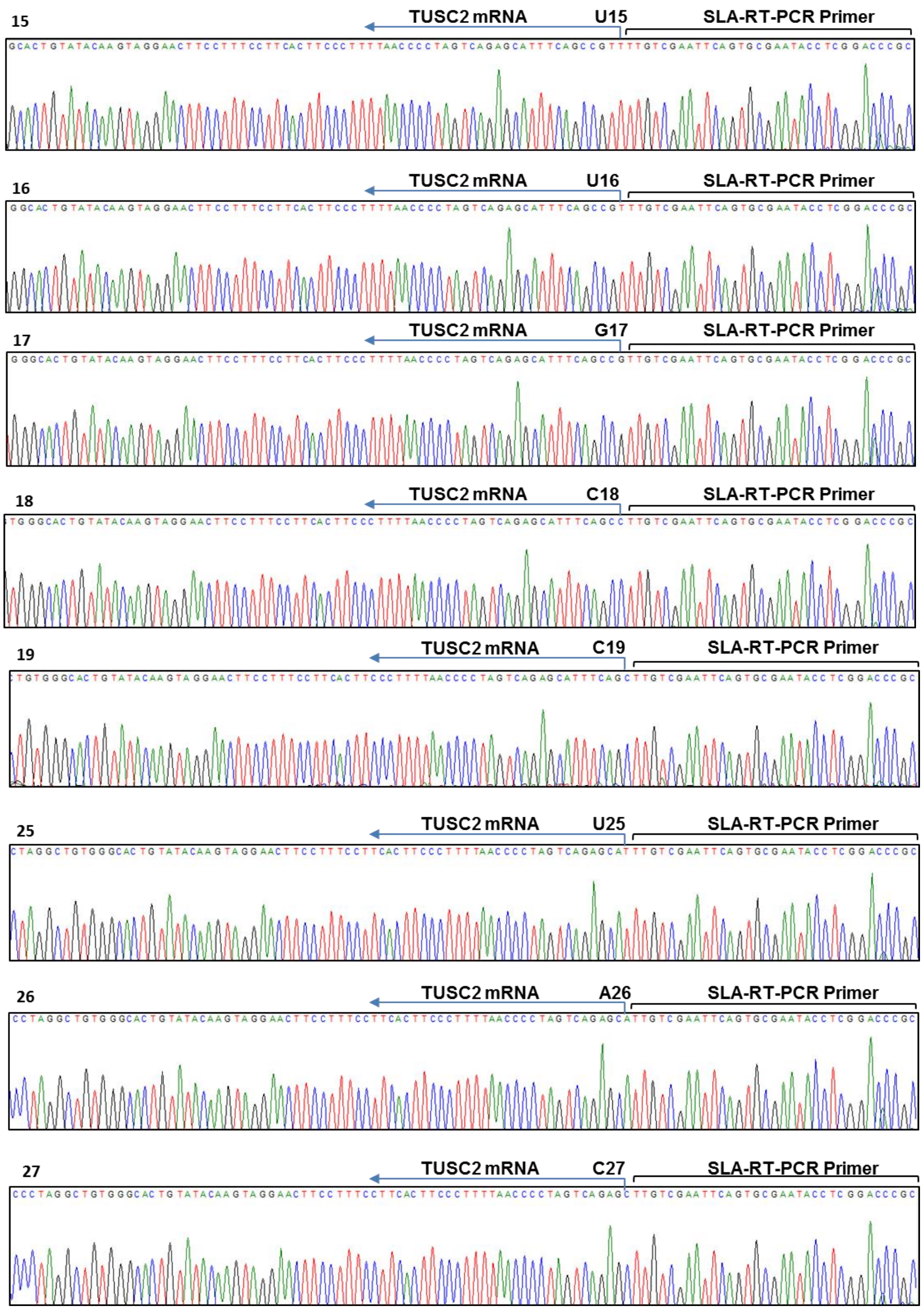

Supplementary Figure. 2. Detection and verification of let-7 miRNA–mediated Cleavage and 3'-uridylation Activities on *TUSC2* target mRNA in H1299 cells by nU-SLA–qRT-PCR Assay and automated DNA sequencing.

(**a**) The sequence alignments of let-7 miRNA:*TUSC2* mRNA target.

(**b**) Relative fragment abundance (RFA) of the cleaved and 3'-uridylated *TUSC2* mRNA fragments at selected bases detected by qRT-PCR with nU-SLA-RT primers. Up to 15 uridines added to the 3'-termini of the cleaved mRNA fragments were readily detectable.

(**c**) The verification of selected cleavage sites and 3'-uridylated target mRNA fragments detected by RT-PCR with 2U-SLA-RT primers as presented in (**b**, **2Us**) and **Figure 1a.** The sequencing profiles of cleaved and 3'-uridylated mRNA fragments at each corresponding nucleotide position on TUSC2 mRNA sequence were coordinately indicated.

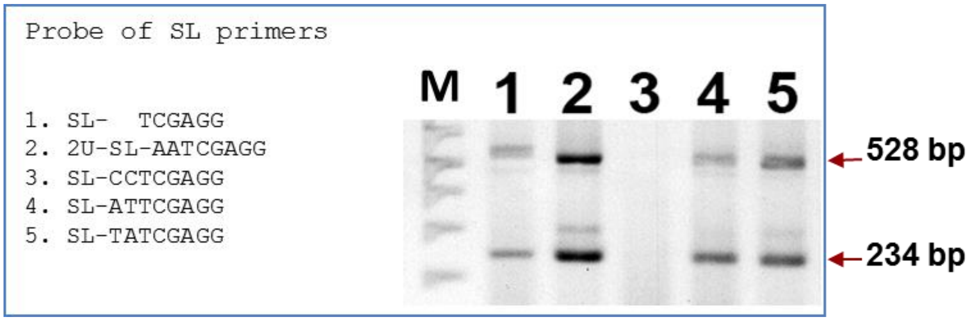

Supplementary Figure 3. Priming Efficiency of the SLA-RT and 2U-SLA-RT Primers. The priming specificities of SL-RT primers with different nt between probes and SL were tested with the A4 fragments of the pLJ-T214 transcript, which produced strong SLA–RT-PCR amplicons of 528 bp and 234 bp at the T1 and T2 target sites, respectively. Substitutes CC, AT and TA were used in place of AA nt. The lane numbers correspond to the SLA-RT primer used. A universal PCR primer and pLJ-T214 transcript-specific primers were used for end-point PCR amplification. SLA–RT-PCR and 2U-SLA–RT-PCR products were analyzed by 2% agarose gel electrophoresis. M: 0.03 µg of 1-kb DNA ladder.

Quantitative Real-time RT-PCR (qRT-PCR) Primer and Probe sequences for all figures

Figure 2a. kRAS-specifc qPCR primers and TaqMan probe:
Sense primer: 5’-TGGAATTCCTTTTATTGAAACATCAG-3’
Antisense primer: 5’-GTGCGGGTCCGAGGTATTC-3’ (universal PCR primer based on SL-RT primer sequence)
TaqMan probe sequence: 5’- GACAGGGTGTTGATGATGCCTTCTATACA-3’

Figure 4b. qPCR primers for detection of ST2 cleavage activity in H1299 transfected by pLJ-T214 with SYBR green real-time PCR:
Sense primer: 5’-CTAGCTAAAGGACTGACCGGCA-3’
Antisense primer: 5’-GTGCGGGTCCGAGGTATTC-3’ (universal PCR primer based on SL-RT primer sequence)

Figure 4d. qPCR primers and TaqMan probe for detection ST2 cleavage activity in H1299 transfected with pLJ-T722 plasmid:
Sense primer: 5’- CGGCATGGACGAGCTGTACAAGTA-3’
Antisense primer: 5’-GTGCGGGTCCGAGGTATTC-3’ (universal PCR primer based on SL-RT primer sequence)
TaqMan probe: 5’-CTGACCGGCAAGTTGGACGCCCGCAAGA-3’
qPCR primers for detection of both ST1 and ST2 (AT1+ST2) activities in H1299 cells transfected by pLJ-T722 plasmid with SYBR green real-time qPCR:
Sense primer: 5’- CGGCATGGACGAGCTGTACAAGTA-3’

Antisense primer: 5’-GTGCGGGTCCGAGGTATTC-3’ (universal PCR primer based on SL-RT primer sequence).

Supplementary Table 1: SLA-RT Primers and PCR Primers for Detection of let-7-cleaved *TUSC2* 5' mRNA Fragments

1a. SLA-RT primers and PCR primers for *TUSC2* mRNA fragment detection

Primer Number	Bases	Stem loop array RT probe (5'–3')	Detected 3'-terminal sequences of TUSC2 Target mRNA (3'-5')
		**hsa-miR-98 5'- UGAGGUAGUAAGU---UGUAUUGUU -3'**	
		|||||||:||:: || | ||:|	
		**TUSC2 mRNA 3'- CCUCCUUAGCUCCAUCGUUUGCCGACUUUACGAGACUGAUC -5'**	
		||||||	
1	C1	GTCGAATTCAGTGCGGGTCCGAGGTATTCGCACTGAATTCGAC GAATCG	CUUAGCUCCAU......
2	U2	GTCGAATTCAGTGCGGGTCCGAGGTATTCGCACTGAATTCGAC AATCGA	UUAGCUCCAUC......
3	U3	GTCGAATTCAGTGCGGGTCCGAGGTATTCGCACTGAATTCGAC ATCGAG	UAGCUCCAUCG......
4	A4	GTCGAATTCAGTGCGGGTCCGAGGTATTCGCACTGAATTCGAC TCGAGG	AGCUCCAUCGU......
5	G5	GTCGAATTCAGTGCGGGTCCGAGGTATTCGCACTGAATTCGAC CGAGGT	GCUCCAUCGUU......
6	C6	GTCGAATTCAGTGCGGGTCCGAGGTATTCGCACTGAATTCGAC GAGGTA	CUCCAUCGUUU......
7	U7	GTCGAATTCAGTGCGGGTCCGAGGTATTCGCACTGAATTCGAC AGGTAG (b)	UCCAUCGUUUG......
8	C8	GTCGAATTCAGTGCGGGTCCGAGGTATTCGCACTGAATTCGAC GGTAGC	CCAUCGUUUGC......
9	C9	GTCGAATTCAGTGCGGGTCCGAGGTATTCGCACTGAATTCGAC GTAGCA	CAUCGUUUGCC......
10	A10	GTCGAATTCAGTGCGGGTCCGAGGTATTCGCACTGAATTCGAC TAGCAA	AUCGUUUGCCG......
11	U11	GTCGAATTCAGTGCGGGTCCGAGGTATTCGCACTGAATTCGAC AGCAAA (c)	UCGUUUGCCGA......
12	C12	GTCGAATTCAGTGCGGGTCCGAGGTATTCGCACTGAATTCGAC GCAAAC (d)	CGUUUGCCGAC......
13	G13	GTCGAATTCAGTGCGGGTCCGAGGTATTCGCACTGAATTCGAC CAAACG	GUUUGCCGACU......
14	U14	GTCGAATTCAGTGCGGGTCCGAGGTATTCGCACTGAATTCGAC AAACGG	UUUGCCGACUU......
15	U15	GTCGAATTCAGTGCGGGTCCGAGGTATTCGCACTGAATTCGAC AACGGC	UUGCCGACUUU......
16	U16	GTCGAATTCAGTGCGGGTCCGAGGTATTCGCACTGAATTCGAC ACGGCT	UGCCGACUUUA......
17	G17	GTCGAATTCAGTGCGGGTCCGAGGTATTCGCACTGAATTCGAC CGGCTG	GCCGACUUUAC......
18	C18	GTCGAATTCAGTGCGGGTCCGAGGTATTCGCACTGAATTCGAC GGCTGA	CCGACUUUACG......
19	C19	GTCGAATTCAGTGCGGGTCCGAGGTATTCGCACTGAATTCGAC GCTGAA	CGACUUUACGA......
20	G20	GTCGAATTCAGTGCGGGTCCGAGGTATTCGCACTGAATTCGAC CTGAAA	GACUUUACGAG......
21	A21	GTCGAATTCAGTGCGGGTCCGAGGTATTCGCACTGAATTCGAC TGAAAT	ACUUUACGAGA......
22	C22	GTCGAATTCAGTGCGGGTCCGAGGTATTCGCACTGAATTCGAC GAAATG	CUUUACGAGAC......
23	U23	GTCGAATTCAGTGCGGGTCCGAGGTATTCGCACTGAATTCGAC AAATGC	UUUACGAGACU......
24	U24	GTCGAATTCAGTGCGGGTCCGAGGTATTCGCACTGAATTCGAC AATGCT	UUACGAGACUG......
25	U25	GTCGAATTCAGTGCGGGTCCGAGGTATTCGCACTGAATTCGAC ATGCTC	UACGAGACUGA......
26	A26	GTCGAATTCAGTGCGGGTCCGAGGTATTCGCACTGAATTCGAC TGCTCT	ACGAGACUGAU......
27	C27	GTCGAATTCAGTGCGGGTCCGAGGTATTCGCACTGAATTCGAC GCTCTG	CGAGACUGAUC......
28	G28	GTCGAATTCAGTGCGGGTCCGAGGTATTCGCACTGAATTCGAC CTCTGA	GAGACUGAUCC......
29	A29	GTCGAATTCAGTGCGGGTCCGAGGTATTCGCACTGAATTCGAC TCTGAC	AGACUGAUCCC......
30	G30	GTCGAATTCAGTGCGGGTCCGAGGTATTCGCACTGAATTCGAC CTGACT	GACUGAUCCCC......
31	A31	GTCGAATTCAGTGCGGGTCCGAGGTATTCGCACTGAATTCGAC TGACTA	ACUGAUCCCCA......
32	C32	GTCGAATTCAGTGCGGGTCCGAGGTATTCGCACTGAATTCGAC GACTAG	CUGAUCCCCAA......

Universal PCR primer: 5'- GTGCGGGTCCGAGGTATTC -3'
Stem Loop sequence: 5'- GTCGAATTCAGTGCGGGTCCGAGGTATTCGCACTGAATTCGAC -3'
TUSC2 mRNA-specific PCR primer: 5'- TACCTGGTTCCTTGCCTGGT -3'
pLJ-T214 transcript-specific PCR primer: 5'- CGGCATGGACGAGCTGTACAAGTA -3'

(a): The SL-RT primer for C32 was replaced with SL-CACAGC with 311 bp and 75 bp amplicons expected respectively for pLJ-T214 Transcript T2 and T1 sites in Figure 3a.
(b): This primer primes an additional fragment of 290 bp on TUSC2 mRNA.
(c): This primer primes an additional fragment of 332 bp on TUSC2 mRNA.
(d): This primer primes an additional fragment of 333 bp on TUSC2 mRNA.

1b. 2U-SLA-RT primers and PCR primers for *TUSC2* mRNA fragment detection

Primer Number	Bases	Stem loop array RT probe (5'–3')	Detected 3'-terminal sequences of TUSC2 Target mRNA (3'-5')
		hsa-miR-98 5'- UGAGGUAGUAAGU---UGUAUUGUU -3'	
		|||||||:||:: || | ||:|	
		TUSC2 mRNA 3'- CCUCCUUAGCUCCAUCGUUUGCCGACUUUACGAGACUGAUC -5'	
		||||||	
1	C1	GTCGAATTCAGTGCGGGTCCGAGGTATTCGCACTGAATTCGAC AA GAATCG	UUCUUAGCUCC......
2	U2	GTCGAATTCAGTGCGGGTCCGAGGTATTCGCACTGAATTCGAC AA AATCGA	UUUUAGCUCCA......
3	U3	GTCGAATTCAGTGCGGGTCCGAGGTATTCGCACTGAATTCGAC AA ATCGAG	UUUAGCUCCAU......
4	A4	GTCGAATTCAGTGCGGGTCCGAGGTATTCGCACTGAATTCGAC AA TCGAGG	UUAGCUCCAUC......
5	G5	GTCGAATTCAGTGCGGGTCCGAGGTATTCGCACTGAATTCGAC AA CGAGGT	UUGCUCCAUCG......
6	C6	GTCGAATTCAGTGCGGGTCCGAGGTATTCGCACTGAATTCGAC AA GAGGTA	UUCUCCAUCGU......
7	U7	GTCGAATTCAGTGCGGGTCCGAGGTATTCGCACTGAATTCGAC AA AGGTAG (a)	UUUCCAUCGUU......
8	C8	GTCGAATTCAGTGCGGGTCCGAGGTATTCGCACTGAATTCGAC AA GGTAGC	UUCCAUCGUUU......
9	C9	GTCGAATTCAGTGCGGGTCCGAGGTATTCGCACTGAATTCGAC AA GTAGCA	UUCAUCGUUUG......
10	A10	GTCGAATTCAGTGCGGGTCCGAGGTATTCGCACTGAATTCGAC AA TAGCAA	UUAUCGUUUGC......
11	U11	GTCGAATTCAGTGCGGGTCCGAGGTATTCGCACTGAATTCGAC AA AGCAAA (b)	UUUCGUUUGCC......
12	C12	GTCGAATTCAGTGCGGGTCCGAGGTATTCGCACTGAATTCGAC AA GCAAAC (c)	UUCGUUUGCCG......
13	G13	GTCGAATTCAGTGCGGGTCCGAGGTATTCGCACTGAATTCGAC AA CAAACG	UUGUUUGCCGA......
14	U14	GTCGAATTCAGTGCGGGTCCGAGGTATTCGCACTGAATTCGAC AA AAACGG	UUUUUGCCGAC......
15	U15	GTCGAATTCAGTGCGGGTCCGAGGTATTCGCACTGAATTCGAC AA AACGGC	UUUUGCCGACU......
16	U16	GTCGAATTCAGTGCGGGTCCGAGGTATTCGCACTGAATTCGAC AA ACGGCT	UUUGCCGACUU......
17	G17	GTCGAATTCAGTGCGGGTCCGAGGTATTCGCACTGAATTCGAC AA CGGCTG	UUGCCGACUUU......
18	C18	GTCGAATTCAGTGCGGGTCCGAGGTATTCGCACTGAATTCGAC AA GGCTGA	UUCCGACUUUA......
19	C19	GTCGAATTCAGTGCGGGTCCGAGGTATTCGCACTGAATTCGAC AA GCTGAA	UUCGACUUUAC......
20	G20	GTCGAATTCAGTGCGGGTCCGAGGTATTCGCACTGAATTCGAC AA CTGAAA	UUGACUUUACG......
21	A21	GTCGAATTCAGTGCGGGTCCGAGGTATTCGCACTGAATTCGAC AA TGAAAT	UUACUUUACGA......
22	C22	GTCGAATTCAGTGCGGGTCCGAGGTATTCGCACTGAATTCGAC AA GAAATG	UUCUUUACGAG......
23	U23	GTCGAATTCAGTGCGGGTCCGAGGTATTCGCACTGAATTCGAC AA AAATGC	UUUUUACGAGA......
24	U24	GTCGAATTCAGTGCGGGTCCGAGGTATTCGCACTGAATTCGAC AA AATGCT	UUUUACGAGAC......
25	U25	GTCGAATTCAGTGCGGGTCCGAGGTATTCGCACTGAATTCGAC AA ATGCTC	UUUACGAGACU......
26	A26	GTCGAATTCAGTGCGGGTCCGAGGTATTCGCACTGAATTCGAC AA TGCTCT	UUACGAGACUG......
27	C27	GTCGAATTCAGTGCGGGTCCGAGGTATTCGCACTGAATTCGAC AA GCTCTG	UUCGAGACUGA......
28	G28	GTCGAATTCAGTGCGGGTCCGAGGTATTCGCACTGAATTCGAC AA CTCTGA	UUGAGACUGAU......
29	A29	GTCGAATTCAGTGCGGGTCCGAGGTATTCGCACTGAATTCGAC AA TCTGAC	UUAGACUGAUC......
30	G30	GTCGAATTCAGTGCGGGTCCGAGGTATTCGCACTGAATTCGAC AA CTGACT	UUGACUGAUCC......
31	A31	GTCGAATTCAGTGCGGGTCCGAGGTATTCGCACTGAATTCGAC AA TGACTA	UUACUGAUCCC......
32	C32	GTCGAATTCAGTGCGGGTCCGAGGTATTCGCACTGAATTCGAC AA GACTAG	UUCUGAUCCCC......

Universal PCR primer: 5'- GTGCGGGTCCGAGGTATTC -3'
Stem Loop sequence: 5'- GTCGAATTCAGTGCGGGTCCGAGGTATTCGCACTGAATTCGAC -3'
TUSC2 mRNA-specific PCR primer: 5'- TACCTGGTTCCTTGCCTGGT -3'
pLJ-T214 transcript-specific PCR primer: 5'- CGGCATGGACGAGCTGTACAAGTA -3'

(a): This primer primes an additional fragment of 292 bp on TUSC2 mRNA.
(b): This primer primes an additional fragment of 334 bp on TUSC2 mRNA.
(c): This primer primes an additional fragment of 335 bp on TUSC2 mRNA.

1c. SLA-RT primers and PCR primers for pLJ-T722 transcript fragment detection

```
hsa-miR-98        5'-          UGAGGUAGUAAGU---UGUAUUGUU          -3'
                                |||||||:||::   || | ||:|
pLJ-T722 T2       3'-AGUCUAGAUGCUCCAUCGUUUGCCGACUUUACGAGAGCUCGU  -5'
pLJ-T722 T1       3'-UGGAGCAGGGCUCCAUCGUUUGCCGACUUUACGAGACUGAUG  -5'
```

Primer Number	Bases	Stem loop array RT probe (5'–3')	Comment
1	U1	GTCGAATTCAGTGCGGGTCCGAGGTATTCGCACTGAATTCGAC ACCTCG	T1 only
2	A2	GTCGAATTCAGTGCGGGTCCGAGGTATTCGCACTGAATTCGAC TCGTCC	T1 only
3	C3	GTCGAATTCAGTGCGGGTCCGAGGTATTCGCACTGAATTCGAC GTCCCG	T1 only
4	A4	GTCGAATTCAGTGCGGGTCCGAGGTATTCGCACTGAATTCGAC TCCCGA	T1 only
5	G5	GTCGAATTCAGTGCGGGTCCGAGGTATTCGCACTGAATTCGAC CCCGAG	T1 only
6	G6	GTCGAATTCAGTGCGGGTCCGAGGTATTCGCACTGAATTCGAC CCGAGG	T1 only
7	G7	GTCGAATTCAGTGCGGGTCCGAGGTATTCGCACTGAATTCGAC CGAGGT	T1 and T2
8	C8	GTCGAATTCAGTGCGGGTCCGAGGTATTCGCACTGAATTCGAC GAGGTA	T1 and T2
9	U9	GTCGAATTCAGTGCGGGTCCGAGGTATTCGCACTGAATTCGAC AGGTAG	T1 and T2
10	C10	GTCGAATTCAGTGCGGGTCCGAGGTATTCGCACTGAATTCGAC GGTAGC	T1 and T2
11	C11	GTCGAATTCAGTGCGGGTCCGAGGTATTCGCACTGAATTCGAC GTAGCA	T1 and T2
12	A12	GTCGAATTCAGTGCGGGTCCGAGGTATTCGCACTGAATTCGAC TAGCAA	T1 and T2
13	U13	GTCGAATTCAGTGCGGGTCCGAGGTATTCGCACTGAATTCGAC AGCAAA	T1 and T2
14	C14	GTCGAATTCAGTGCGGGTCCGAGGTATTCGCACTGAATTCGAC GCAAAC	T1 and T2
15	G15	GTCGAATTCAGTGCGGGTCCGAGGTATTCGCACTGAATTCGAC CAAACG	T1 and T2
16	U16	GTCGAATTCAGTGCGGGTCCGAGGTATTCGCACTGAATTCGAC AAACGG	T1 and T2
17	U17	GTCGAATTCAGTGCGGGTCCGAGGTATTCGCACTGAATTCGAC ACGGCT	T1 and T2
18	C18	GTCGAATTCAGTGCGGGTCCGAGGTATTCGCACTGAATTCGAC GGCTGA	T1 and T2
19	G19	GTCGAATTCAGTGCGGGTCCGAGGTATTCGCACTGAATTCGAC CTGAAA	T1 and T2
20	C20	GTCGAATTCAGTGCGGGTCCGAGGTATTCGCACTGAATTCGAC GAAATG	T1 and T2
21	U21	GTCGAATTCAGTGCGGGTCCGAGGTATTCGCACTGAATTCGAC AATGCT	T1 and T2
22	U22	GTCGAATTCAGTGCGGGTCCGAGGTATTCGCACTGAATTCGAC ATGCTC	T1 and T2
23	A23	GTCGAATTCAGTGCGGGTCCGAGGTATTCGCACTGAATTCGAC TGCTCT	T1 and T2
24	C24	GTCGAATTCAGTGCGGGTCCGAGGTATTCGCACTGAATTCGAC GCTCTG	T1 only
25	G25	GTCGAATTCAGTGCGGGTCCGAGGTATTCGCACTGAATTCGAC CTCTGA	T1 and T2 (a)
26	A26	GTCGAATTCAGTGCGGGTCCGAGGTATTCGCACTGAATTCGAC TCTGAC	T1 and T2 (a)
27	G27	GTCGAATTCAGTGCGGGTCCGAGGTATTCGCACTGAATTCGAC CTGACT	T1 and T2 (a)
28	A28	GTCGAATTCAGTGCGGGTCCGAGGTATTCGCACTGAATTCGAC TGACTA	T1 only
29	D50	GTCGAATTCAGTGCGGGTCCGAGGTATTCGCACTGAATTCGAC cgccaa	T1 and T2 (b)
30	D50	GTCGAATTCAGTGCGGGTCCGAGGTATTCGCACTGAATTCGAC gccaca	T1 and T2 (c)
31	D50	GTCGAATTCAGTGCGGGTCCGAGGTATTCGCACTGAATTCGAC gatctt	T2 only (d)
32	D50	GTCGAATTCAGTGCGGGTCCGAGGTATTCGCACTGAATTCGAC cgtccc	T2 only (e)

Universal PCR primer: 5'- GTGCGGGTCCGAGGTATTC -3'
Stem Loop sequence: 5'- GTCGAATTCAGTGCGGGTCCGAGGTATTCGCACTGAATTCGAC -3'
pLJ-T722 transcript-specific PCR primer: 5'- CGGCATGGACGAGCTGTACAAGTA -3'

(a): Identical sequences were located at 53 nt down at 5' end of T2 site.
(b): Identical target sequences were located 60 nt down to the 5' end of T1 or T2 sites respectively.
(c): Identical target sequences were located 110 nt down to the 5' end of T1 or T2 sites. Multiple targets were located on the transcripts.
(d): Identical target sequences were located 217 nt down to the 5' end of T2 site.
(e): Identical target sequences were located 261 nt down to the 5' end of T2 site.

1d. SLA-RT primers and PCR primers for *k-RAS*:miR-622 target fragment detection

<table>
<tr><th rowspan="5">Primer Number</th><th rowspan="5">Bases</th><th colspan="2">SLA-RT Primer (5'–3')</th></tr>
<tr><td>hsa-miR-622</td><td>5'- ACAGUCUGC-UGAGGUUGGAGC</td></tr>
<tr><td></td><td>||||||||| || ||| |||:</td></tr>
<tr><td>kRAS mRNA</td><td>3'-UUUCGAUUGUCAGACGUACCUCGUCCUUUUUUUAAU-5'</td></tr>
<tr><td></td><td>||||||</td></tr>
<tr><td>1</td><td>C1</td><td colspan="2">GTCGAATTCAGTGCGGGTCCGAGGTATTCGCACTGAATTCGAC GCTAAC</td></tr>
<tr><td>2</td><td>G2</td><td colspan="2">GTCGAATTCAGTGCGGGTCCGAGGTATTCGCACTGAATTCGAC CTAACA</td></tr>
<tr><td>3</td><td>A3</td><td colspan="2">GTCGAATTCAGTGCGGGTCCGAGGTATTCGCACTGAATTCGAC TAACAG</td></tr>
<tr><td>4</td><td>U4</td><td colspan="2">GTCGAATTCAGTGCGGGTCCGAGGTATTCGCACTGAATTCGAC AACAGT</td></tr>
<tr><td>5</td><td>U5</td><td colspan="2">GTCGAATTCAGTGCGGGTCCGAGGTATTCGCACTGAATTCGAC ACAGTC</td></tr>
<tr><td>6</td><td>G6</td><td colspan="2">GTCGAATTCAGTGCGGGTCCGAGGTATTCGCACTGAATTCGAC CAGTCT</td></tr>
<tr><td>7</td><td>U7</td><td colspan="2">GTCGAATTCAGTGCGGGTCCGAGGTATTCGCACTGAATTCGAC AGTCTG</td></tr>
<tr><td>8</td><td>C8</td><td colspan="2">GTCGAATTCAGTGCGGGTCCGAGGTATTCGCACTGAATTCGAC GTCTGC</td></tr>
<tr><td>9</td><td>A9</td><td colspan="2">GTCGAATTCAGTGCGGGTCCGAGGTATTCGCACTGAATTCGAC TCTGCA</td></tr>
<tr><td>10</td><td>G10</td><td colspan="2">GTCGAATTCAGTGCGGGTCCGAGGTATTCGCACTGAATTCGAC CTGCAT</td></tr>
<tr><td>11</td><td>A11</td><td colspan="2">GTCGAATTCAGTGCGGGTCCGAGGTATTCGCACTGAATTCGAC TGCATG</td></tr>
<tr><td>12</td><td>C12</td><td colspan="2">GTCGAATTCAGTGCGGGTCCGAGGTATTCGCACTGAATTCGAC GCATGG</td></tr>
<tr><td>13</td><td>G13</td><td colspan="2">GTCGAATTCAGTGCGGGTCCGAGGTATTCGCACTGAATTCGAC CATGGA</td></tr>
<tr><td>14</td><td>U14</td><td colspan="2">GTCGAATTCAGTGCGGGTCCGAGGTATTCGCACTGAATTCGAC ATGGAG</td></tr>
<tr><td>15</td><td>A15</td><td colspan="2">GTCGAATTCAGTGCGGGTCCGAGGTATTCGCACTGAATTCGAC TGGAGC</td></tr>
<tr><td>16</td><td>C16</td><td colspan="2">GTCGAATTCAGTGCGGGTCCGAGGTATTCGCACTGAATTCGAC GGAGCA</td></tr>
<tr><td>17</td><td>C17</td><td colspan="2">GTCGAATTCAGTGCGGGTCCGAGGTATTCGCACTGAATTCGAC GAGCAG</td></tr>
<tr><td>18</td><td>U18</td><td colspan="2">GTCGAATTCAGTGCGGGTCCGAGGTATTCGCACTGAATTCGAC AGCAGG</td></tr>
<tr><td>19</td><td>C19</td><td colspan="2">GTCGAATTCAGTGCGGGTCCGAGGTATTCGCACTGAATTCGAC GCAGGA</td></tr>
<tr><td>20</td><td>G20</td><td colspan="2">GTCGAATTCAGTGCGGGTCCGAGGTATTCGCACTGAATTCGAC CAGGAA</td></tr>
<tr><td>21</td><td>U21</td><td colspan="2">GTCGAATTCAGTGCGGGTCCGAGGTATTCGCACTGAATTCGAC AGGAAA</td></tr>
<tr><td>22</td><td>C22</td><td colspan="2">GTCGAATTCAGTGCGGGTCCGAGGTATTCGCACTGAATTCGAC GGAAAA</td></tr>
<tr><td colspan="4">Universal PCR primer: 5'- GTGCGGGTCCGAGGTATTC -3'
Stem Loop sequence: 5'- GTCGAATTCAGTGCGGGTCCGAGGTATTCGCACTGAATTCGAC -3'
Ki-ras mRNA specific PCR primer: 5'- TGGAATTCCTTTTATTGAAACATCAG -3'</td></tr>
</table>

1e. 2U-SLA-RT primers and PCR primers for *k-RAS*:miR-622 target fragment detection

Primer Number	Bases	Stem loop array 2U RT probe (5'–3')
		hsa-miR-622 5'- ACAGUCUGC-UGAGGUUGGAGC -3'
		|||||||| || ||| |||:
		k-RAS mRNA 3'-UUUCGAUUGUCAGACGUACCUCGUCCUUUUUUUAAU-5'
		||||||
1	C1	GTCGAATTCAGTGCGGGTCCGAGGTATTCGCACTGAATTCGAC AA GCTAAC
2	G2	GTCGAATTCAGTGCGGGTCCGAGGTATTCGCACTGAATTCGAC AA CTAACA
3	A3	GTCGAATTCAGTGCGGGTCCGAGGTATTCGCACTGAATTCGAC AA TAACAG
4	U4	GTCGAATTCAGTGCGGGTCCGAGGTATTCGCACTGAATTCGAC AA AACAGT
5	U5	GTCGAATTCAGTGCGGGTCCGAGGTATTCGCACTGAATTCGAC AA ACAGTC
6	G6	GTCGAATTCAGTGCGGGTCCGAGGTATTCGCACTGAATTCGAC AA CAGTCT
7	U7	GTCGAATTCAGTGCGGGTCCGAGGTATTCGCACTGAATTCGAC AA AGTCTG
8	C8	GTCGAATTCAGTGCGGGTCCGAGGTATTCGCACTGAATTCGAC AA GTCTGC
9	A9	GTCGAATTCAGTGCGGGTCCGAGGTATTCGCACTGAATTCGAC AA TCTGCA
10	G10	GTCGAATTCAGTGCGGGTCCGAGGTATTCGCACTGAATTCGAC AA CTGCAT
11	A11	GTCGAATTCAGTGCGGGTCCGAGGTATTCGCACTGAATTCGAC AA TGCATG
12	C12	GTCGAATTCAGTGCGGGTCCGAGGTATTCGCACTGAATTCGAC AA GCATGG
13	G13	GTCGAATTCAGTGCGGGTCCGAGGTATTCGCACTGAATTCGAC AA CATGGA
14	U14	GTCGAATTCAGTGCGGGTCCGAGGTATTCGCACTGAATTCGAC AA ATGGAG
15	A15	GTCGAATTCAGTGCGGGTCCGAGGTATTCGCACTGAATTCGAC AA TGGAGC
16	C16	GTCGAATTCAGTGCGGGTCCGAGGTATTCGCACTGAATTCGAC AA GGAGCA
17	C17	GTCGAATTCAGTGCGGGTCCGAGGTATTCGCACTGAATTCGAC AA GAGCAG
18	U18	GTCGAATTCAGTGCGGGTCCGAGGTATTCGCACTGAATTCGAC AA AGCAGG
19	C19	GTCGAATTCAGTGCGGGTCCGAGGTATTCGCACTGAATTCGAC AA GCAGGA
20	G20	GTCGAATTCAGTGCGGGTCCGAGGTATTCGCACTGAATTCGAC AA CAGGAA
21	U21	GTCGAATTCAGTGCGGGTCCGAGGTATTCGCACTGAATTCGAC AA AGGAAA
22	C22	GTCGAATTCAGTGCGGGTCCGAGGTATTCGCACTGAATTCGAC AA GGAAAA

Universal PCR primer: 5'- GTGCGGGTCCGAGGTATTC -3'
Stem Loop sequence: 5'- GTCGAATTCAGTGCGGGTCCGAGGTATTCGCACTGAATTCGAC -3'
Ki-ras mRNA specific PCR primer: 5'- TGGAATTCCTTTTATTGAAACATCAG -3'

1f. 8U-SLA-RT primers and PCR primers for *kRAS*:miR-622 target fragment detection

```
hsa-miR-622     5'-         ACAGUCUGC-UGAGGUUGGAGC        -3'
                             |||||||| || ||| |||:
k-RAS mRNA      3'-UUUCGAUUGUCAGACGUACCUCGUCCUUUUUUUAAU-5'
                        ||||||
```

Primer Number	Bases	Stem loop array 8U RT probe (5'–3')
1	C1	GTCGAATTCAGTGCGGGTCCGAGGTATTCGCACTGAATTCGAC AAAAAAAA GCTAAC
2	G2	GTCGAATTCAGTGCGGGTCCGAGGTATTCGCACTGAATTCGAC AAAAAAAA CTAACA
3	A3	GTCGAATTCAGTGCGGGTCCGAGGTATTCGCACTGAATTCGAC AAAAAAAA TAACAG
4	U4	GTCGAATTCAGTGCGGGTCCGAGGTATTCGCACTGAATTCGAC AAAAAAAA AACAGT
5	U5	GTCGAATTCAGTGCGGGTCCGAGGTATTCGCACTGAATTCGAC AAAAAAAA ACAGTC
6	G6	GTCGAATTCAGTGCGGGTCCGAGGTATTCGCACTGAATTCGAC AAAAAAAA CAGTCT
7	U7	GTCGAATTCAGTGCGGGTCCGAGGTATTCGCACTGAATTCGAC AAAAAAAA AGTCTG
8	C8	GTCGAATTCAGTGCGGGTCCGAGGTATTCGCACTGAATTCGAC AAAAAAAA GTCTGC
9	A9	GTCGAATTCAGTGCGGGTCCGAGGTATTCGCACTGAATTCGAC AAAAAAAA TCTGCA
10	G10	GTCGAATTCAGTGCGGGTCCGAGGTATTCGCACTGAATTCGAC AAAAAAAA CTGCAT
11	A11	GTCGAATTCAGTGCGGGTCCGAGGTATTCGCACTGAATTCGAC AAAAAAAA TGCATG
12	C12	GTCGAATTCAGTGCGGGTCCGAGGTATTCGCACTGAATTCGAC AAAAAAAA GCATGG
13	G13	GTCGAATTCAGTGCGGGTCCGAGGTATTCGCACTGAATTCGAC AAAAAAAA CATGGA
14	U14	GTCGAATTCAGTGCGGGTCCGAGGTATTCGCACTGAATTCGAC AAAAAAAA ATGGAG
15	A15	GTCGAATTCAGTGCGGGTCCGAGGTATTCGCACTGAATTCGAC AAAAAAAA TGGAGC
16	C16	GTCGAATTCAGTGCGGGTCCGAGGTATTCGCACTGAATTCGAC AAAAAAAA GGAGCA
17	C17	GTCGAATTCAGTGCGGGTCCGAGGTATTCGCACTGAATTCGAC AAAAAAAA GAGCAG
18	U18	GTCGAATTCAGTGCGGGTCCGAGGTATTCGCACTGAATTCGAC AAAAAAAA AGCAGG
19	C19	GTCGAATTCAGTGCGGGTCCGAGGTATTCGCACTGAATTCGAC AAAAAAAA GCAGGA
20	G20	GTCGAATTCAGTGCGGGTCCGAGGTATTCGCACTGAATTCGAC AAAAAAAA CAGGAA
21	U21	GTCGAATTCAGTGCGGGTCCGAGGTATTCGCACTGAATTCGAC AAAAAAAA AGGAAA
22	C22	GTCGAATTCAGTGCGGGTCCGAGGTATTCGCACTGAATTCGAC AAAAAAAA GGAAAA

Universal PCR primer: 5'- GTGCGGGTCCGAGGTATTC -3'
Stem Loop sequence: 5'- GTCGAATTCAGTGCGGGTCCGAGGTATTCGCACTGAATTCGAC -3'
Ki-ras mRNA specific PCR primer: 5'- TGGAATTCCTTTTATTGAAACATCAG -3'

1g. SLA-RT primers and PCR primers for detection of cleaved pLJ-T214 transcript fragments with AGO2 knockdown

<table>
<tr><th rowspan="2">Primer Number</th><th rowspan="2">Bases</th><th>Stem loop RT probe (5'–3')</th><th rowspan="2">Detected 3'-terminal sequences of pLJ-T214 Target mRNA (3'-5')</th></tr>
<tr><td>hsa-miR-98 5′- UGAGGUAGUAAGU---UGUAUUGUU -3′
|||||||:||: || | ||:|
pLJ-T214 3′- CCUCCUUAGCUCCAUCGUUUGCCGACUUUACGAGACUGAUC -5′
||||||</td></tr>
<tr><td>1</td><td>A4</td><td>GTCGAATTCAGTGCGGGTCCGAGGTATTCGCACTGAATTCGAC TCGAGG</td><td>AGCUCCAUCGU......</td></tr>
<tr><td>2</td><td>G17</td><td>GTCGAATTCAGTGCGGGTCCGAGGTATTCGCACTGAATTCGAC CGGCTG</td><td>GCCGACUUUAC......</td></tr>
<tr><td>3</td><td>C18</td><td>GTCGAATTCAGTGCGGGTCCGAGGTATTCGCACTGAATTCGAC GGCTGA</td><td>CCGACUUUACG......</td></tr>
<tr><td>4</td><td>C27</td><td>GTCGAATTCAGTGCGGGTCCGAGGTATTCGCACTGAATTCGAC GCTCTG</td><td>CGAGACUGAUC......</td></tr>
<tr><td>5</td><td>A4</td><td>GTCGAATTCAGTGCGGGTCCGAGGTATTCGCACTGAATTCGAC AA TCGAGG</td><td>UUAGCUCCAUCGU......</td></tr>
<tr><td>6</td><td>G17</td><td>GTCGAATTCAGTGCGGGTCCGAGGTATTCGCACTGAATTCGAC AA CGGCTG</td><td>UUGCCGACUUUAC......</td></tr>
<tr><td>7</td><td>C18</td><td>GTCGAATTCAGTGCGGGTCCGAGGTATTCGCACTGAATTCGAC AA GGCTGA</td><td>UUCCGACUUUACG......</td></tr>
<tr><td>8</td><td>C27</td><td>GTCGAATTCAGTGCGGGTCCGAGGTATTCGCACTGAATTCGAC AA GCTCTG</td><td>UUCGAGACUGAUC......</td></tr>
<tr><td colspan="4">Universal PCR primer: 5′- GTGCGGGTCCGAGGTATTC -3′
Stem Loop sequence: 5′- GTCGAATTCAGTGCGGGTCCGAGGTATTCGCACTGAATTCGAC -3′
pLJ-T214 transcript-specific PCR primer: 5′- CGGCATGGACGAGCTGTACAAGTA -3′</td></tr>
</table>

Supplementary Table 2. Sequences of TUTase-siRNAs and SL-RT-PCR Primers

2a. TUTase-siRNA sequences

Target	Sequence
**TUTase-1**	5′-CGAGCACAUUCACUAACAA-3′
**TUTase-2**	5′-CGUUAGUGCUGGUGAUUAA-3′
**TUTase-3**	5′-GGACGACACUUCAAUUAUU-3′
**TUTase-3**	5′-GGACGACACUUCAAUUA -3′
**TUTase-5**	5′-CUACGGUACCAAUAAUAAA-3′
**U6 TUTase**	5′-GCAGCCAAUUACUGCCGAA-3′
**TUTase-7**	5′-GAAAAGAGGCACAAGAAAA-3

2b. SL-RT primers for detection of TUTase mRNA expression

Target	Sequence
**TUTase-2**	sense: 5′-GCAGACTTGTCTAGAGCTGTG -3′
	antisense: 5′-CTCGAATCAGCTGAGGTCTCTC-3′
**TUTase-3**	sense: 5′-GAGTAACAGATGAAGTTGCCAC -3′
	antisense: 5′-GTTTGAGTTGTACCTTGGAAGC-3′
Universal PCR primer: 5′- GTGCGGGTCCGAGGTATTC -3′<br>Stem Loop sequence:<br>5′- GTCGAATTCAGTGCGGGTCCGAGGTATTCGCACTGAATTCGAC -3′	

Supplementary Table 3. SL-RT Primers and PCR Primers for miRNA Detection

miRNA	miRNA-specific PCR primer	mature miRNA sequence	Stem loop RT probe
hsa-let-7a	CGAAGCTTGAGGTAGTAGGTTGT	ugagguaguagguuguauaguu	GTCGAATTCAGTGCGGGTCCGAGGTATTCGCACTGAATTCGAC AACTAT
hsa-let-7b	CGAAGCTTGAGGTAGTAGGTTGT	ugagguaguagguugugugguu	GTCGAATTCAGTGCGGGTCCGAGGTATTCGCACTGAATTCGAC AACCAC
hsa-let-7c	CGAAGCTTGAGGTAGTAGGTTGT	ugagguaguagguuguaugguu	GTCGAATTCAGTGCGGGTCCGAGGTATTCGCACTGAATTCGAC AACCAT
hsa-let-7d	CGAAGCTAGAGGTAGTAGGTTGC	agagguaguagguugcauaguu	GTCGAATTCAGTGCGGGTCCGAGGTATTCGCACTGAATTCGAC AACTAT
Has-let-7e	CGAAGCTTGAGGTAGGAGGTTGT	ugagguaggagguuguauaguu	GTCGAATTCAGTGCGGGTCCGAGGTATTCGCACTGAATTCGAC AACTAT
hsa-let-7f	CGAAGCTTGAGGTAGTAGATTGT	ugagguaguagauuguauaguu	GTCGAATTCAGTGCGGGTCCGAGGTATTCGCACTGAATTCGAC AACTAT
hsa-let-7g	CGAAGCTTGAGGTAGTAGTTTGT	ugagguaguaguuuguacaguu	GTCGAATTCAGTGCGGGTCCGAGGTATTCGCACTGAATTCGAC AACTGT
hsa-let-7i	CGAAGCTTGAGGTAGTAGTTTGT	ugagguaguaguuugugcuguu	GTCGAATTCAGTGCGGGTCCGAGGTATTCGCACTGAATTCGAC AACAGC
hsa-miR-98	CGAAGCTTGAGGTAGTAAGTTGTATTG	ugagguaguaaguuguauuguu	GTCGAATTCAGTGCGGGTCCGAGGTATTCGCACTGAATTCGAC AACAAT
hsa-miR-30a	ATTGAAGCTTGTAAACATCCTCGACTGG	uguaaacauccucgacuggaag	GTCGAATTCAGTGCGGGTCCGAGGTATTCGCACTGAATTCGAC CTTCCA
hsa-miR-622	CGAAGCTACAGTCTGCTGAGGTTG	acagucugcugagguuggagc	GTCGAATTCAGTGCGGGTCCGAGGTATTCGCACTGAATTCGAC GCTCCA
hsa-RNU44	CCTGGATGATGATAAGCAAATGC	ccuggaugaugauaagcaaaugc...acugacu	GTCGAATTCAGTGCGGGTCCGAGGTATTCGCACTGAATTCGAC AGTCAG
Universal PCR primer: 5’- GTGCGGGTCCGAGGTATTC -3’			
Stem Loop sequence: 5’- GTCGAATTCAGTGCGGGTCCGAGGTATTCGCACTGAATTCGAC -3’			
